# Supplementary material for: Kinetically controlled metal-elastomer nanophases for environmentally resilient stretchable electronics
Source: Nat Commun. 2024 Apr 9;15:3071. doi: 10.1038/s41467-024-47223-6 (PMC11004024; doi:10.1038/s41467-024-47223-6)
Supplement: Supplementary file 1 — Supplementary Information [file 41467_2024_47223_MOESM1_ESM.docx]

Supplementary Information for

**Kinetically controlled metal-elastomer nanophases for environmentally resilient stretchable electronics**

*Soosang Chae, Won Jin Choi*, Lisa Julia Nebel, Chang Hee Cho, Quinn A. Besford, André Knapp, Oliver Sander, Pavlo Makushko, Yevhen Zabila, Oleksandr Pylypovskyi, Min Woo Jeong, Stanislav Avdoshenko, Denys Makarov, Yoon Jang Chung, Andreas Fery, Jin Young Oh* and Tae Il Lee**

Correspondence to:

Won Jin Choi, email: [choi21@llnl.gov](mailto:choi21@llnl.gov),

Tae Il Lee, email: [t2.lee77@gachon.ac.kr](mailto:t2.lee77@gachon.ac.kr),

Jin Young Oh, email: [jyoh@khu.ac.kr](mailto:jyoh@khu.ac.kr)

**This PDF file includes**

Suppl. Notes 1 to 8

Suppl. Figures 1 to 26

Suppl. Tables 1 to 3

Suppl. References

**Other Suppl. Information for this manuscript include the following:**

Supplementary Movies 1 to 6

**Suppl. Note 1. Analytical mechanics of gyrification**

The gyrification index (GI) is a metric used to quantify the level of gyrification of a surface, and we have adopted this concept here to compare our structural features of 3D complex of nanophase with other wrinkles/buckles. We have specifically focused on two parameters: the amplitude of wrinkles and the number of wrinkles per 100 µm. As one might expect, higher GI values are achieved with greater numbers and larger amplitudes of wrinkles. We denote the amplitude as "*x*" and the number per 100 µm as "*y*," with examples represented as (*x*, *y*) (Suppl. Figs. 6a–d).

However, attaining a high GI value is not a straightforward task due to the trade-off correlation between wrinkle amplitudes and their density. This negative correlation becomes evident when examining the equations for wavelength (*λ*) and amplitude (*A*) of wrinkles, as outlined below:

$\lambda=2\pi h{(\bar{E_{p}}/3\bar{E_{m}})}^{1/3} (1)$

$A = h\sqrt{\varepsilon/\varepsilon_{c}-1}$ (2)

where $h$ is the film thickness, $\bar{E}=E/(1-\nu^{2})$ is the plane-strain elastic modulus, $E$ is the elastic modulus, $\nu$ is the Poisson’s ratio, $\varepsilon_{c}=\frac{1}{4}{(3\bar{E_{m}}/\bar{E_{p}})}^{2/3}$ is the critical wrinkling strain, $\varepsilon$ is the compressive strain^1^.

Here, the subscripts p and m indicate PDMS substrate and metallic thin film, respectively. We found that both $\lambda$ and *A* are proportional to $\bar{E_{p}}/(3\bar{E_{m}})$; thus, the number of wrinkles per 100 µm (*y*) is inversely proportional to this value as plotted in Suppl. Fig. 6e. Here, the ratio $\bar{E_{p}}/(3\bar{E_{m}})$ essentially reflects the elastic modulus relationship between the metal film and PDMS substrate. It directly suggests that, to achieve a high GI value, the value of $\bar{E_{p}}/(3\bar{E_{m}})$ should neither be too low nor too high, as shown in Suppl. Fig. 6e. However, achieving this desired value is challenging since it requires the elastic modulus of the metallic film to be comparable to that of PDMS. In the case of conventional metallic films like Au, which have a modulus of around tens of GPa, and PDMS with a modulus of several MPa, obtaining the necessary GI value becomes unfeasible due to the substantial difference in elastic modulus^2,3^. Gyrified structures are indeed distinct from existing wrinkles, as clearly demonstrated in Suppl. Fig. 6f. Highlighting the key aspects, the attainment of highly gyrified structures (GI >1.6) has not been achievable through conventional methods. Nanoblending emerges as a pivotal technique in transcending the trade-off between wrinkle amplitude and density by modulating the modulus.

**Suppl. Note 2. Optical analysis of Au-PDMS nanophase**

The nanophase starts to grow with Au nanoparticles close to spheres at the beginning of deposition and thickens with Au nanoneedles as deposition progresses. An electromagnetic (EM) study of the nanosphere and nanoneedle provides further insight on the broadening of the absorbance spectrum. The results indicate that when the shape of a particle is a sphere, the peak positions are not changed by the radius until it grows from 1 nm to 20 nm (Suppl. Fig. 7c–e). Only the amplitudes of peaks increase by a factor of 5. Because the ratio between peaks and baseline is also changed by the radius, color change possibly occurs with the growth of the nanosphere; however, according to HRTEM analysis, the structure of Au nanostructures changes from spheres to needles for reticular formation. This may be a well-known phenomenon, but an EM study was conducted based on the dimensions of nanoneedles obtained from the TEM analysis (Suppl. Fig. 7f–h). As the shape of Au nanostructures changed from nanosphere and nanorod to nanoneedle, the main LSPR peaks exhibit a red shift (Suppl. Fig. 7g). LSPR depends strongly on the length (*l*) of the nanostructure, and in general, the peak of resonance is proportional to the *l* of the Au structures based on the lumped circuit model. The resonance peak can be defined as *f* = 1/2π(*LC*)^1/2^, where *L* and *C* are the inductance and capacitance of the Au nanostructure^4^. Since both of *L* and *C* are proportional to the *l* of Au nanoneedle, then *f* is inversely proportional to *l*; thus, the wavelength of LSPR is proportional to *l*. Similar to the case in nanospheres, the magnitude of scattering cross-section for Au nanoneedles increases as *l* increases (Suppl. Figs. 7h).

**Suppl. Note 3. Formation Mechanism of Au-PDMS nanophases.**

While Au deposition on PDMS with *φ* = 10 results in typical thin-films of Au layers, PDMS with *φ* < 10 no longer provides a conducive surface for the free diffusion of Au atoms. This is due to the interaction between Si-H groups in the excess crosslinker and Au atoms, leading to the formation of Si-Au chemical bonds. Excess crosslinkers are distributed in an equilibrium concentration throughout the volume and surface of PDMS when prepared under *φ* < 10 conditions. As *φ* decreases, the absolute concentration of these excess linkers increases.

When Au nucleates on the PDMS surface and forms small nanoparticles, the crosslinkers occupy the interface between Au nanoparticles and PDMS, resulting in the formation of Si-Au bonds. This phenomenon can be understood in terms of surface energy: the introduction of an Au nanoparticle onto PDMS replaces the original PDMS/vacuum interface with new PDMS/Au and Au/vacuum interfaces. Given that the interfacial energy of PDMS/vacuum (19.00 mN m^-1^) is lower than that of Au/vacuum (1,500 mN m^-1^), the system seeks to minimize the overall surface energy. Consequently, the system favors the presence of the Au/vacuum interface over that of the PDMS/vacuum interface.

Driven by this energy minimization principle, the excess crosslinkers spontaneously migrate to the surface of the Au nanoparticle, leading to the creation of Si-Au bonds. As illustrated schematically in Suppl. Fig. 8a, the migration of crosslinkers can be visualized as occurring upward along the surface of the Au nanoparticle from the triple junction involving Au, PDMS, and vacuum.

Here, let's consider a scenario where the deposition of Au is ongoing while the crosslinker is undergoing migration. When Au atoms are introduced onto the substrate, they do not adhere to the regions already occupied by the crosslinker. Instead, they settle onto the surfaces of Au nanoparticles that have not yet been covered by the migrating crosslinkers. This process facilitates the growth of the Au nanoparticles.

In cases where the migration flux of the crosslinker is significantly faster than the deposition flux of Au, the Au nanoparticle becomes completely enveloped by the crosslinker, preventing further growth. As a result, any additional incoming Au atoms can only lead to the nucleation and growth of secondary Au nanoparticles on other sites of the PDMS surface. These secondary Au nanoparticles quickly become coated with crosslinkers once again (Suppl. Fig. 8b). This mechanism elucidates the formation process of particulate nanophases.

However, when the deposition flux of Au is comparable to the migration flux of the crosslinker, continuous growth of Au occurs near the upper surface of the Au nanoparticle that remains unoccupied by the crosslinker. Concurrently, the crosslinkers continue their migration along the newly grown Au surface (Suppl. Fig. 8c). This mechanism corresponds to the formation process of the reticular nanophase.

To validate the nanophase formation mechanisms discussed above, we conducted observations of microstructures in nanophases based on varying deposition fluxes of Au, as presented in Suppl. Fig. 9. As the deposition flux increases, a nanophase consisting of progressively longer Au nanoneedles emerges, providing support for our proposed reticular nanophase formation mechanism.

Drawing upon our experimental findings, we have formulated a concise summary of the mechanism underlying nanophase formation as follows: (I) Under conditions where the migration rate of the excess crosslinker significantly exceeds the deposition rate of Au, the resulting nanophase takes on the form of discrete particles; (II) When these two rates are approximately equal, a nanophase characterized by a reticular structure is generated; and (III) In cases where the deposition rate greatly surpasses the migration rate of the excess crosslinker, the outcome is a straightforward deposition of a metal thin film, devoid of nanophase formation.

**Suppl. Note 4. Plain PDMS membrane mechanical characterization for simulation**

To get simulations of labyrinth 3D complex, a simulation model based on real hyperelastic substrate behavior was necessary. This simulation required a more detailed determination of stress-strain behavior. The PDMS system (Sylgard 184, Dow Corning) allows the modification of the mechanical parameters by changes of the component ratio and by different curing procedures. The characterization was done with uniaxial tensile tests for different component ratios with 5-6 single specimens to get the stress-strain relationship. Every tensile raw data includes the measured specimen geometry and measured tensile force $F_{t}$ in N in combination with the elongation way $s_{e}$ in mm. A precise raw data processing was required to get an averaged master stress-strain curve from all specimens for each tested variant of component ratio and curing temperature. This was done with a Python 3.7 script that averaged all single stress-strain curves by stack processing to one averaged master stress-strain curve, which was then used as the basis for mechanical characterization.

The averaging process itself was made between the maximum single start strain value and the mean fracture strain value for all single tests of one variant. This process requires a uniform strain interval, which was not naturally given in the result files. Therefore, all measured points had to be interpolated between existing points to get a global strain interval which is valid for all single test results. The averaging process was done up to the point of the fracture of the first specimen by simple averaging of force values. After that point the first single stress-strain curve ends and a further simple averaging of force values with the remaining stress-strain curves leads mostly to undesired sharp changes in the master stress-strain curve. To avoid exactly this problem, a modified averaging process continued from this point. For the fractured single curve, the force values continually were interpolated with the last slope of this curve before the fracture up to the mean facture strain of all single curves. The values of single strain curves, whose fracture was higher than the mean fracture strain, were ignored for the master stress-strain curve calculation. The complete averaging process is shown in Suppl. Fig. 12.

These resulting master force vs. elongation curves had to be transformed to stress vs. strain curves. Therefore, the initial cross-section area was extracted from the single specimens, averaged, and used for the calculation of the engineering stress, which means that the stress curve was calculated only with the initial cross-section area for the complete elongation range. But every elongated material shows, in reality, a reduced cross-section area depending on the applied force, which underestimates the real stress in the material. By assuming a constant volume of the specimen, the true stress could be calculated with Eq.(3)^5^.

| $A*L=A_{0}*L_{0}\Rightarrow\sigma= \frac{F}{A}=\frac{F*L}{A_{0}*L_{0}}=\sigma_{E}\left( 1+\varepsilon_{E} \right)$ | (3) |
| --- | --- |

The result is a set of master stress-strain curves, which first enabled an easy extraction of the fracture strain, fracture stress and Young’s modulus for every component ratio and curing regime. The Young’s modulus is defined for linear elastic material behavior but depends in the case of hyperplastic material behavior directly on the current strain value.

The hyperelastic constants *C*_ij_ for the selected Mooney-Rivlin-model as hyperelastic material model were done with the FEM software ANSYS 2021R1. For that the different component ratios were implemented in the material database. By curve fitting of the uniaxial stress-strain curves with the selected material model the hyperelastic constants *C*_ij_ could be extracted. The results are summarized in Suppl. Table 1.

**Suppl. Note 5. Computational calculation of 3D complex**

For the numerical finite element simulation, we considered the three layers separately: plain Au layer, Au-PDMS intermediate layer and plain PDMS layer. We modeled the plain PDMS layer as an elastic rectangular block of dimensions of 24 µm $\times$ 24 µm $\times$ 20 µm in *x*-, *y*- and *z*-direction. We used a hyperelastic Mooney-Rivlin material^6^ (Sylgard 184, *φ* = 3.5) with parameters derived from uniaxial tensile tests (see Suppl. Table 1 for the uniaxial tensile tests for Sylgard 184, *φ* = 3.5). For the plain Au layer and the Au-PDMS layer, we used two geometrically exact Cosserat shells as described in the literature^7^. While the shell model is two-dimensional, the thickness of each layer *h*_gold_, *h*_intermediate_ appears as a material parameter. This thickness parameter and the stiffness parameters *µ*_gold_, *λ*_gold_, *µ*_intermediate_, *λ*_intermediate_ of each of the two Cosserat shells determine the elastic behavior of the plain Au layer and the Au-PDMS layer. To model the swelling ratio, we attached a swollen plain Au layer and a swollen Au-PDMS layer to a stretched-out plain PDMS layer. After releasing the stretch on the plain PDMS layer, 3D complex formed due to the stress mismatch. Throughout all simulations, we chose the same maximum penetration depth of Au atoms as in the HRTEM images (Fig. 2a). There was almost no dependence on the deposition thickness in the case of *φ* = 3.5 used in our experiments. Knowing the Young’s modulus for the plain components (plain Au and plain PDMS layer), we conducted different simulations resulting in different surface morphologies with various Young’s moduli (*E*_Y_), swelling ratios of the intermediate layer (*Q*) and various thickness values of plain Au layer (*d*). We suppose both the stiffness (Young’s modulus) of the intermediate layer and the thickness of the plain Au layer to increase when increasing the deposition thickness. Accordingly, we suppose that the swelling ratio of intermediate layer increases along with the stiffening, when increasing the deposition thickness. Under these assumptions, the simulated morphologies matched the experimental 3D complex quite well. We discretized the model using second-order (27-nodes) Lagrange finite elements for the substrate, and second-order (9-nodes) geodesic finite elements for the two shells^8^. As shown experimentally in the literature^9^, no locking occurs for this type of shell discretization. The grid for the substrate is manually graded, with a high resolution in the vicinity of the shells. In total, the substrate grid had 8802 vertices and 64859 degrees of freedom. The attached shell models were discretized on the two-dimensional restriction of the substrate grid to the upper boundary in *z*-direction, which had 2401 vertices. The calculations were done using the DUNE libraries for C++ for solving partial differential equations (PDEs) with grid-based methods^10^.

**Suppl. Note 6. Resistance changes of ideal elastomer and Au-PDMS nanophase conductors on strain**

1) Calculation of the resistance change of an ideal elastic conductor under an applied uniaxial strain. For the one-dimensional deformation of a rectangular-shaped two-terminal electrode pad, the width and thickness are reduced by the Poisson's ratio of the pad during tensile elongation in the longitudinal direction. Even for an ideal elastic conductor that undergoes no change in electrical conductivity under any situation, this shape change results in an increase in the two-terminal resistance of the pad. With the assumption of a perfectly incompressible isotropic material with a Poisson's ratio of 0.5, the two-terminal resistance change in the one-dimensional deformation for the ideal elastic conductor was derived as shown in the Fig. 4c as a cyan solid line (ideal). The detailed arithmetic derivation process is shown in the upper part of Suppl. Fig. 17.

2) Calculation of resistance change of ideal elastic conductor under an applied areal strain. The result is shown in the lower part of Fig. 4c as an orange solid line. The calculation of areal strain for an ideal elastic conductor of a circular disk-shaped pad was derived through computational calculations (see the lower of Suppl. Fig. 17). We assume that the pad consists of a perfectly incompressible isotropic material with a Poisson's ratio of 0.5. As shown in the figure, the resistance across both ends was calculated using the voltage formed on the pad under the condition that a current of 1 A flows at both ends of the diameter.

**Suppl. Note 7. Environmentally resilient performance**

Suppl. Table 2 and 3 show a performance comparison of the 3D complex of Au–PDMS nanophases with a comprehensive survey of previously reported stretchable conductors, which could be categorized in five; 1) carbon and 2) metal nanomaterials embedded in elastomer, such as blending nanomaterials with elastomeric matrix, intrinsically stretchable and electrically conductive 3) liquid metal and 4) organic polymer, and 5) mechanically-guided structural designs on elastomer. Various sets of such materials and methods have been studied for soft electronics and stretchable conducting membranes, which have been mainly focused on achieving high conductivity and mechanical stretchability (Suppl. Table 2). For the practical application of the stretchable conducting membrane into soft electronics, sensors, and actuators, the materials should possess not only strain-invariant high conductivity and mechanical robustness, but also thermal and chemical stability simultaneously (Suppl. Table 3). In general, materials have trade-offs between stabilities against chemicals. Polymers are weak against non-polar organic solvents and could be degraded but are stable under basic and acidic (a wide range of pH) conditions, and vice versa for metals. Meanwhile, mechanically guided structural designs with noble metal can provide chemical stability; however, they are delicate and fragile against mechanical abrasion. The pH stability (primarily within a moderate pH range of 4 – 8) of stretchable electrodes have been addressed in stretchable pH or glucose sensors and mostly relying on encapsulation with silicone or epoxy. The thermal stability of stretchable conductors has been discussed with the potential of stretchable thermal heaters within the operation range of less than 200 °C. Achieving stretchable conductor materials while obtaining multimodal stability simultaneously (including pH, thermal, chemical, and mechanical) remains challenging. As a stretchable conducting membrane, the 3D composite of Au–PDMS nanophases in this study can provide outstanding robustness against various destructive conditions, including a pH range of 2–13, temperatures up to 250 °C, solvents (including water, ethanol, acetone, cyclobenzene, and toluene), mechanical abrasion (up to adhesive of 32 N cm^-1^ and eraser test), and washing by a laundry machine (20 cycles for each 15 min with detergent) as shown in Fig. 5a-d, g. This stability against different environments is attributed to the strongly anchored nanophase inside the elastomer, along with the chemical inertness of the two components, gold and PDMS. This durability allowed us to use the material in practically applicable and reusable hand-motion e-skin devices.

**Suppl. Note 8. VR demonstration of the 3D complex of Au–PDMS nanophases**

Given the exceptional performance of strain-invariant conductivity combined with remarkable robustness beyond the state of the art, we have utilized our materials in potential applications for human motion capture in the style of e-skin applications. We anticipate that this technology will enable low-cost mass production of soft interconnects that can be widely used in bioelectronics, smart wearable devices, and human-machine interfaces for a more immersive experience in extended reality environments. Extended reality, or XR, refers to virtual reality (VR), augmented reality (AR), and mixed reality (MR). Here, we demonstrate that the large change in resistance at constant resistance and low electrical noise enable the easy integration of soft interconnects in XR environments as components of electronic skins for precise and seamless motion tracking. This technology, with its simple wiring and small form factor, can find numerous applications in the design of whole-body tracking systems and haptic devices such as gloves and bodysuits.

To fully illustrate the potential of our Au-PDMS nanophase material as a key player in future bioelectronics, we fabricated a prototype human motion-capture device (Suppl. Fig. 25). In actual operating conditions, we demonstrate large resistance variations at a constant resistivity, coupled with low dynamic electrical noise (20 mΩ per √Hz at a 2.8-Hz cutoff frequency, Suppl. Fig. 26). This technology with straightforward wiring and small form factor can find numerous applications in the design of full-body tracking systems and haptics devices such as gloves and bodysuits. For gesture tracking, we applied 3D complex of Au-PDMS nanophases-based stretchable conductor shaped as narrow ribbons on finger joints (Suppl. Fig. 25, left). With two sensors covering base and middle knuckles, we can precisely reconstruct fingers flexion. This information is transferred to VR compatible environment for real time 3D rendering of flexing of individual fingers and making a fist (Suppl. Fig. 25, right and Suppl. Movie 6). Our 3D complex of Au-PDMS nanophases are attractive as stretchable electrodes and interconnects for applications in soft and bioelectronics and further may find application in a wide range of areas including biomonitoring (within digestive tract), multimodal implantable devices (ocular prosthesis), soft robotics (mechanical strain gated logic gates) and functional fabrics (for space exploration).

Noise characterization: To quantify the electrical noise spectrum of the 3D complex of Au-PDMS nanophases, the sample stripe was electrically connected in a 2-point configuration and its resistance was measured at different driving currents. The measurement was performed in a Tensormeter setup (HZDR Innovation GmbH, Germany) at AC current frequency of 775 Hz and 50 S s^-1^ sampling speed and 60 s measurement window. The noise spectra were obtained by performing a discrete Fourier transform of the measured data (Suppl. Fig. 26).


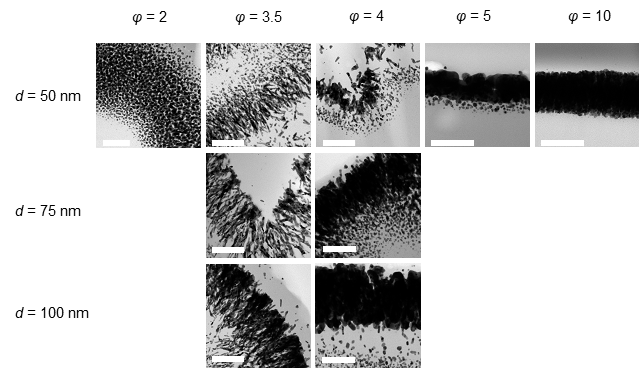


**Suppl. Figure 1.** Cross-section HRTEM images of Au-PDMS nanophases with various *φ* and deposition thickness of Au. Structural variety from particles, elongated nanoparticles, needles, to films was observed. All scale bars denote 200 nm.


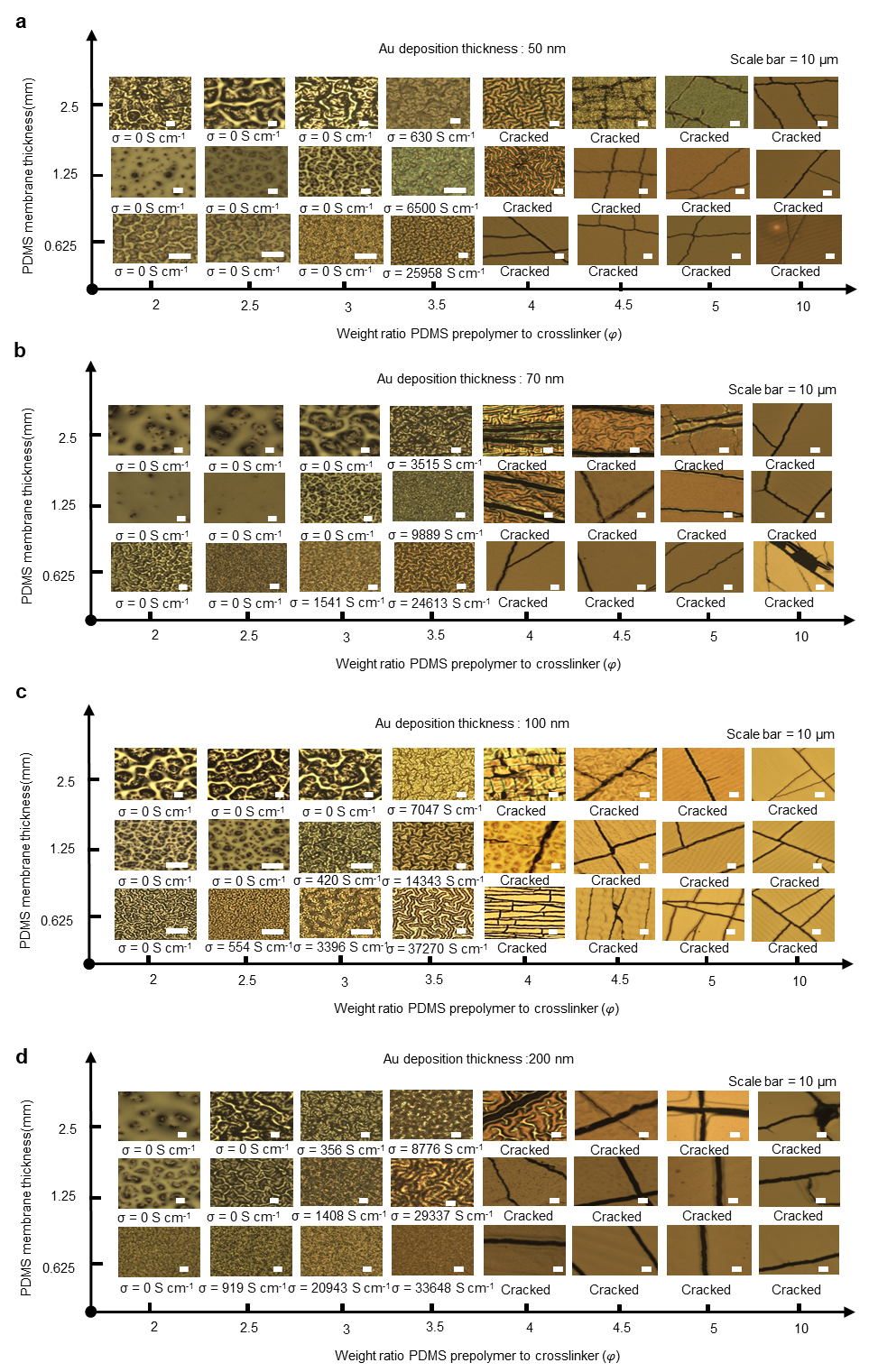


**Suppl. Figure 2.** Surface morphology changes and electrical conductivity of PDMS membranes with deposited Au vary based on the mixing ratio, *φ*, as well as different membrane thicknesses and metal deposition thicknesses: **a**, 50 nm, **b**, 75 nm, **c**, 100 nm, and **d**, 200 nm. The corresponding electrical conductivity, measured using the four-point probe method, also reveals distinct features.


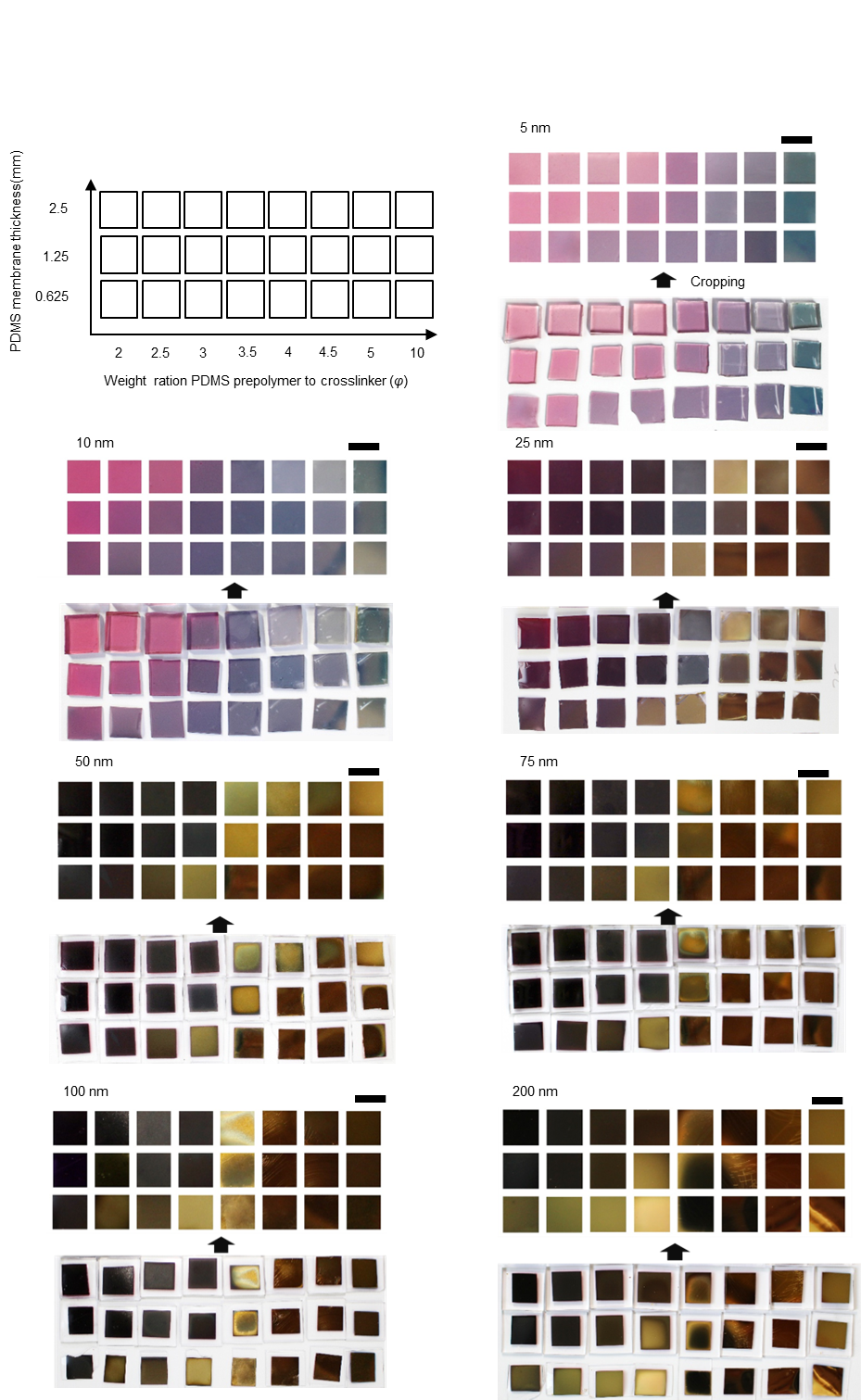


**Suppl. Figure 3.** Variations in Au–PDMS membrane. Photographs showing various colors of Au-deposited PDMS samples with various mixing ratio (*φ*), those membrane thickness, and deposition thickness of the metal. Scale bars denote 1 cm.


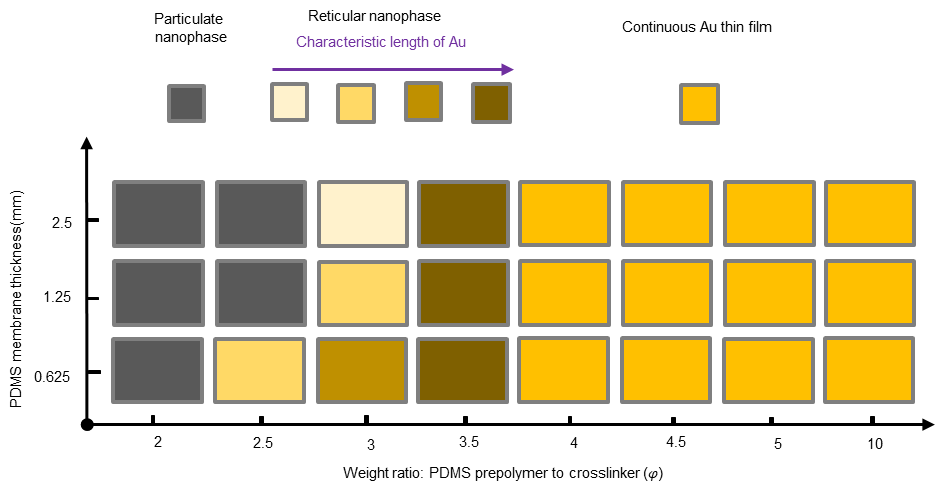


**Suppl. Figure 4.** Classification table of Au-PDMS nanophases based on the process variables; membrane thickness and *φ* at 100 nm of deposition thickness. Conditions in which electrical conductivity could not be measured were classified as having particulate nanophases, and conditions in which electrical conductivity was measured were classified as reticular nanophase.

**
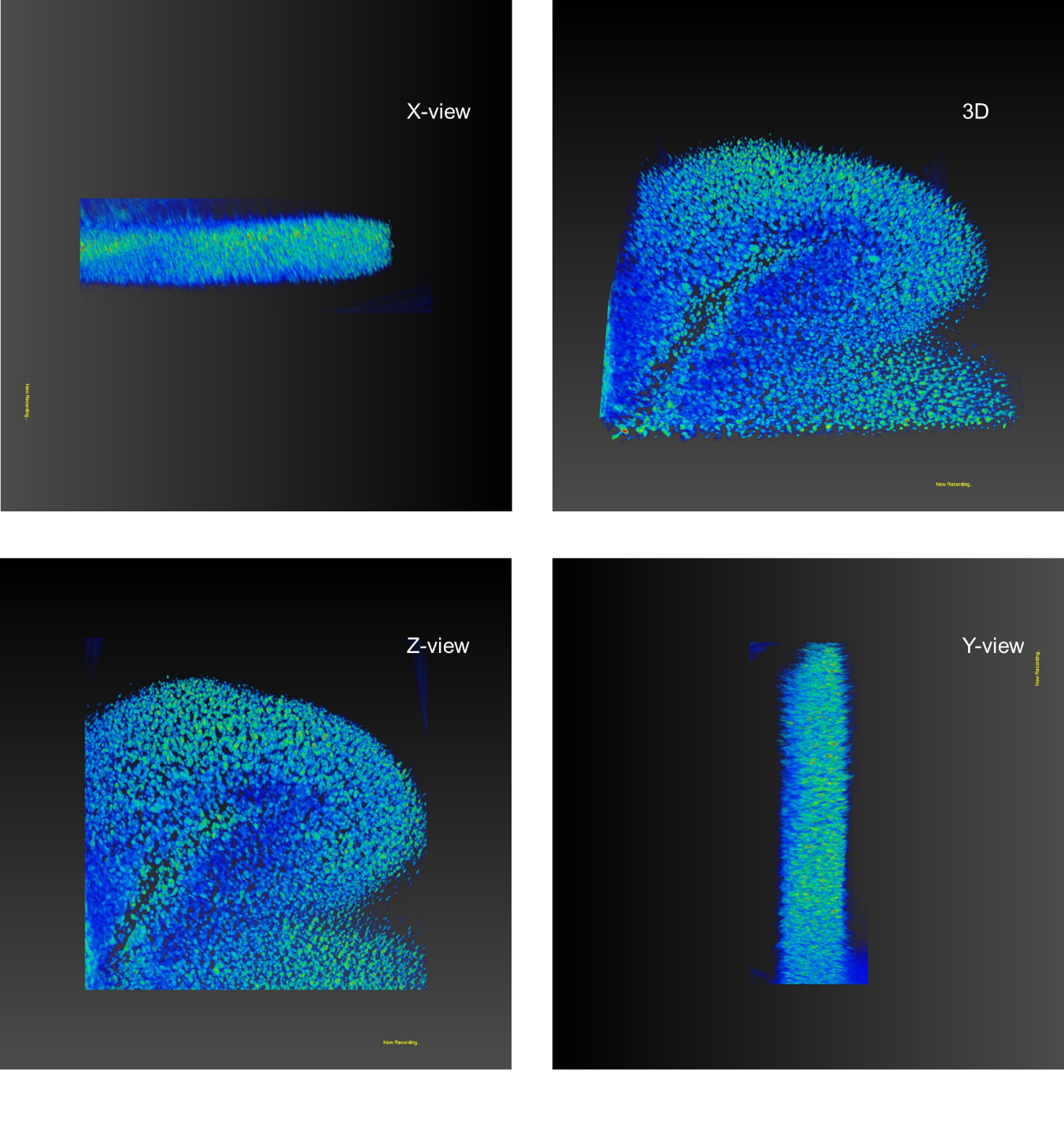
**

**Suppl. Figure 5.** 3D TEM tomography images of Au 100 nm-thick deposited on the PDMS membrane (*φ* = 3.5), showing the interconnected and interpenetrated gyrified metal-elastomer nanophase. The brightness in the tomography images indicates 3D density of Au in the PDMS matrix. Scale bar = 300 nm.


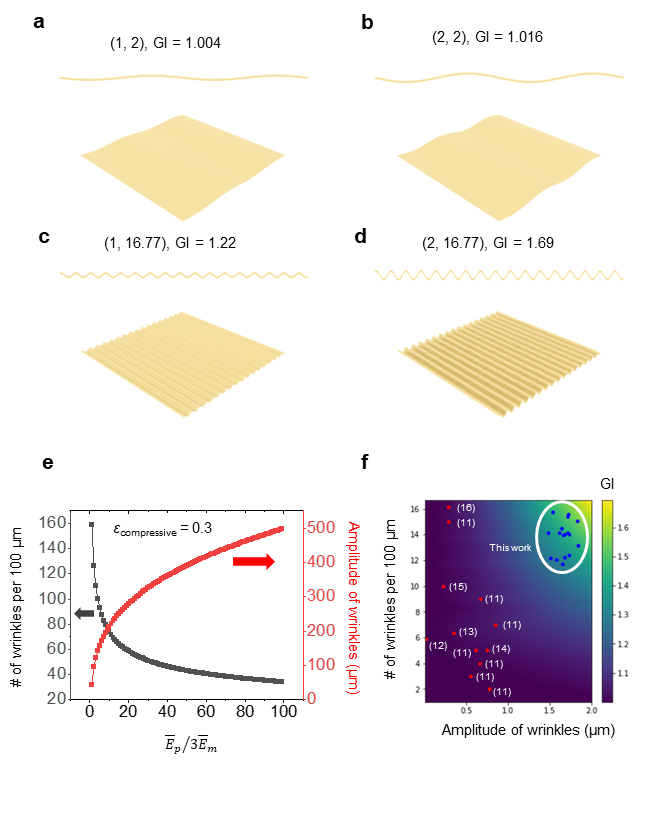


**Suppl. Figure 6. Gyrification index of Au-PDMS nanophase. a**-**d** Examples of wrinkles for coordinated points (1,2), (2,2), (1,16.77), and (2,16.77) in the GI map. **e**, Wrinkles per 100 µm and amplitude of wrinkle as a function of elastic modulus ratio between the metal film and PDMS substrate to show its trade-off behavior. **f**, Map of amplitudes and line density of wrinkles. GI indicates gyrification index and can be obtained by a ratio of the total inner surface area to the area of an outer surface that smoothly encloses the folded/wrinkled surfaces. The amplitudes and the number of wrinkles per 100 μm were extracted from selected Refs. ^11–16^


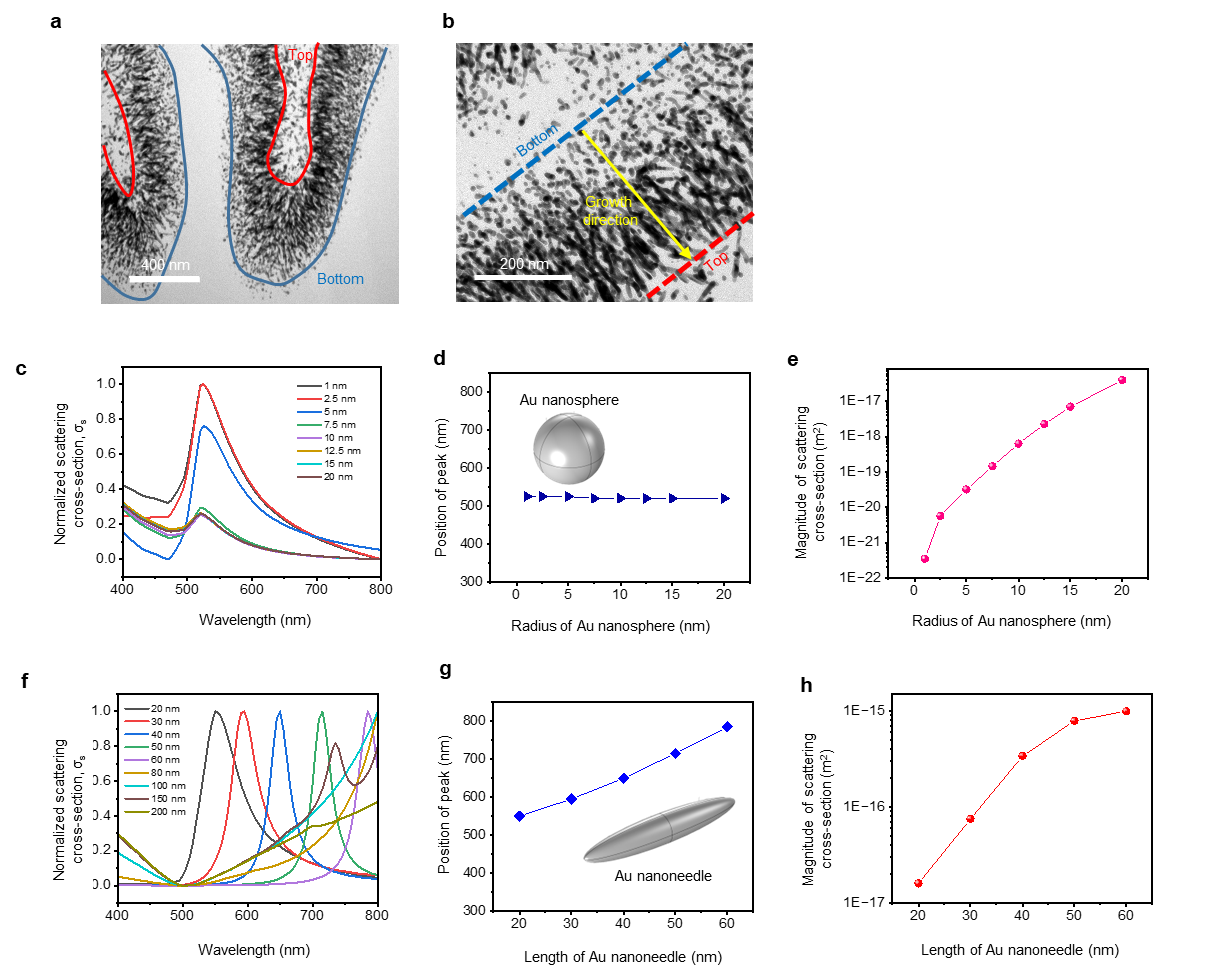


**Suppl. Figure 7. Optical analysis of Au-PDMS nanophase.** **a** and **b** Cross-sectional HRTEM images of a sample (50 nm deposited under the condition of PDMS *φ*=3.5 and 2.5 Å s^-1^). **c**, Calculated normalized scattering cross section as a function of incident electromagnetic wave wavelength, **d**, positions of LSPR peak, and **e**, magnitude of scattering cross section of Au nanospheres with different radiuses. **f**, Calculated normalized scattering cross-section as a function of incident electromagnetic wave wavelength, **g**, positions of LSPR peak, and **h**, magnitude of scattering cross-section of Au nanorods with different lengths. The spectral changes are caused by change of the Au nanostructures, which indicates spherical Au nanoparticles formed at the beginning of nanophase and growth into Au nanoneedles as deposition thickness increases. The origin of the observed peak broadening was well interpreted with the assistance of theoretical electromagnetic calculations. Initially, the LSPR spectrum of the Au nanosphere only appeared at a deposition of 5 nm thickness. As deposition thickness increases (*t* > 7.5 nm thickness), red-shift of LSPR peak was observed due to the superimposition of spectra of Au nanoneedles in addition to those of nanospheres. The TEM images show the length of Au nanoneedles almost linearly increases with respect to the *t*, and the corresponding LSPR spectrum with broad absorbance peak is observed accordingly (*t* = 10 and 20 nm thickness).


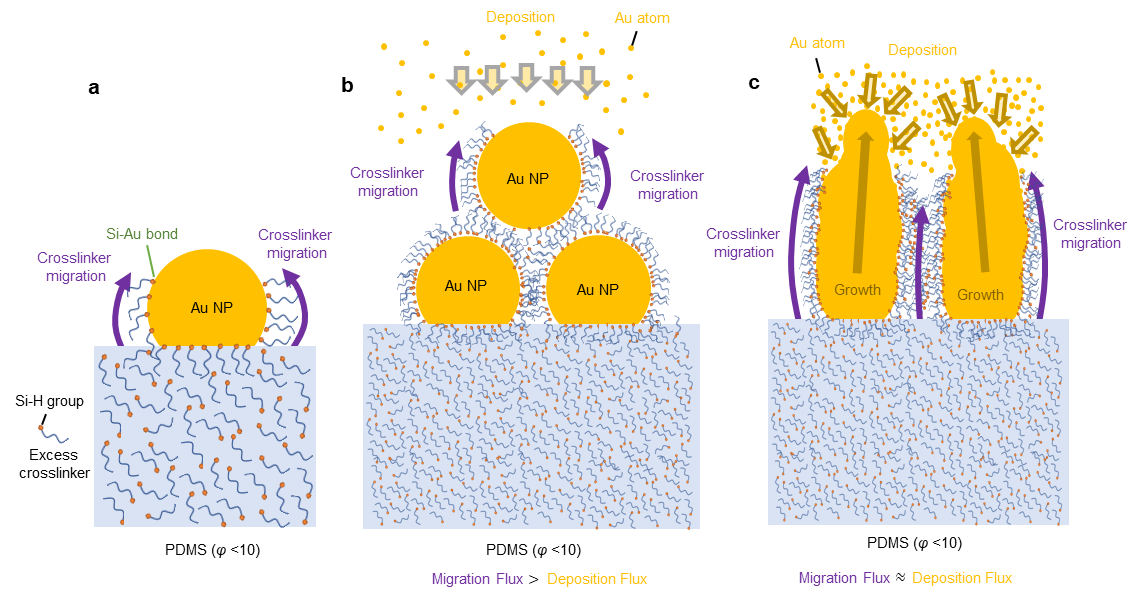


**Suppl. Figure 8. Schemes of growth mechanism on Au-PDMS reticular nanophase.** **a**, Upward migration of the cross-linkers along the surface of the Au nanoparticle. **b**, Particulate nanophase formation process. **c**, Reticular nanophase formation process.


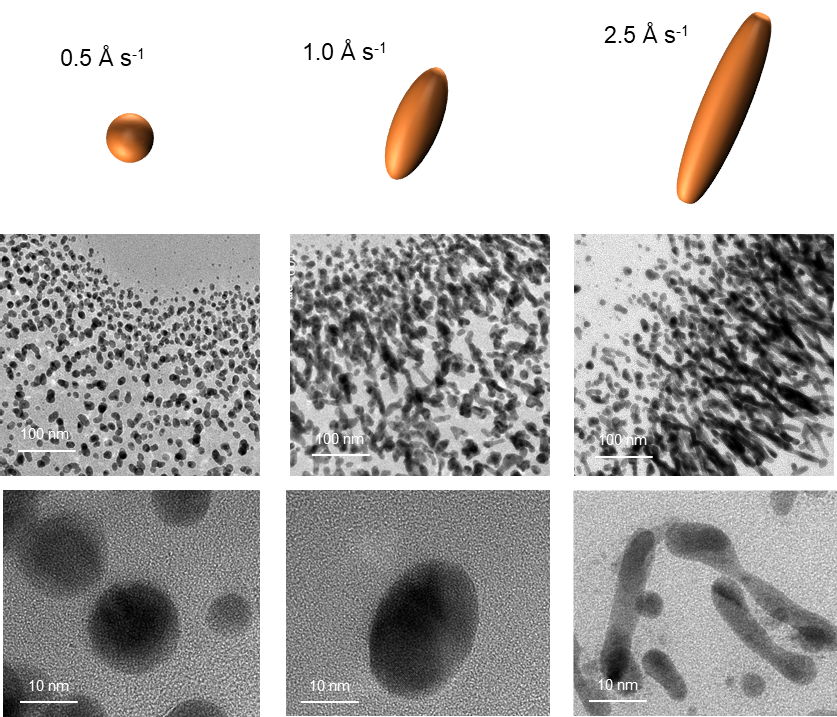


**Suppl. Figure 9. Morphologies of Au-PDMS nanophases.** The samples were investigated with various Au deposition rates (0.5, 1, 2.5 Å s^-1^) at a fixed *φ* of 3.5. The PDMS thickness for Au deposition was set at 1.25 mm and 100 µm, respectively.


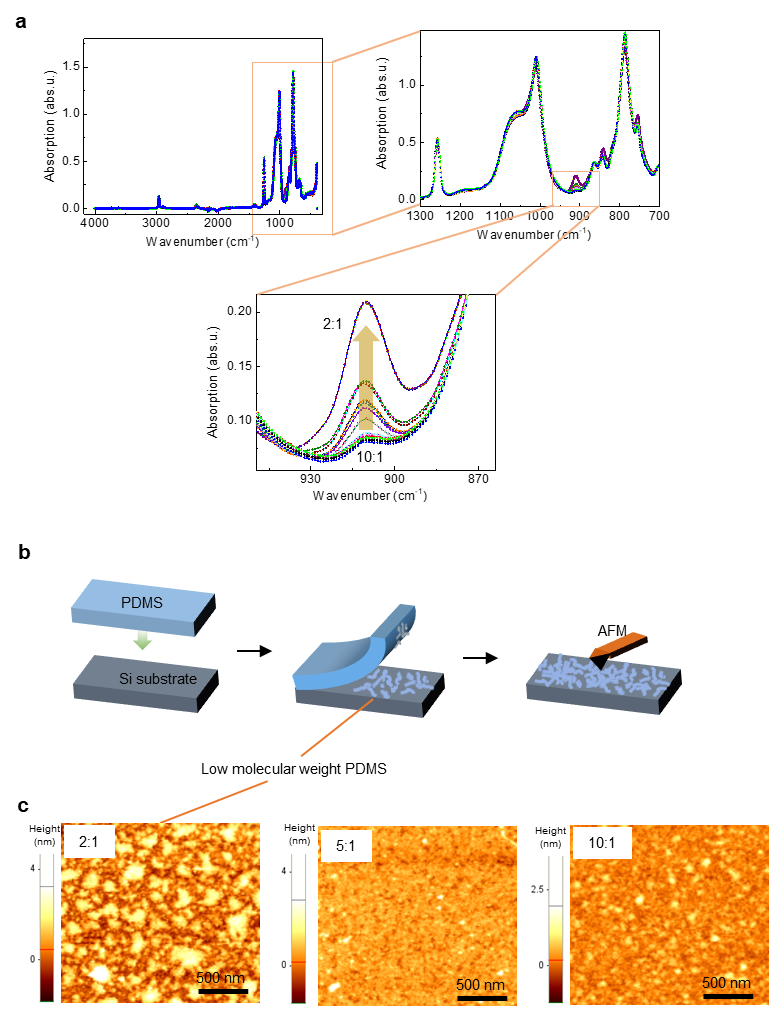


**Suppl. Figure 10. Analysis of uncrosslinked PDMS oligomer. a**, FT-IR analysis of plain PDMS membranes with various curing ratio, *φ*. **b**, Surface uncrosslinked low-molecular weight (*M*w $\approx$ 912,000 g mol^-1^) PDMS and excess PDMS crosslinker was contact printed on Si wafer for 30 min for all PDMS slabs. **c**, AFM images of the contact printed PDMS with various curing ratios.


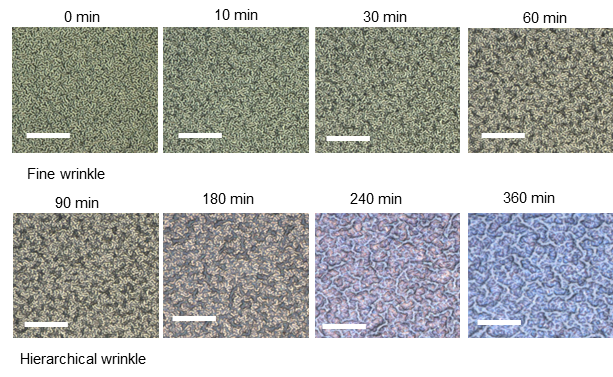


**Suppl. Figure 11.** Laser scanning microscopy (LSM) images of gyrification of Au-PDMS nanophases at *φ* = 2.0 and 2.5 Å s^-1^. The evolution of gyrification at *φ* = 2.0 occurs more rapidly compared to that at *φ* = 3.5 resulting in distinct morphologies (as depicted in Fig. 3a). Scale bars correspond to 10 μm.


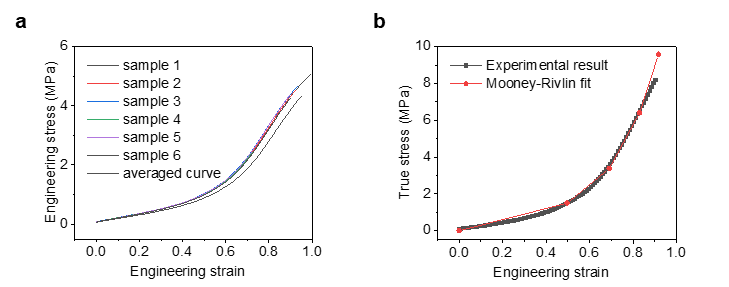


**Suppl. Figure 12. Mechanical characteristics of PDMS for FEM simulation. a**, Measured stress-strain curves for five tested 3.5:1 PDMS samples according to the testing standard DIN 53504/S2/30 with different fracture strains for the single samples and the resulting averaged stress-strain curve. **b**, Comparison between the experimental finding and the FEM simulation, with the parameters specified in Supplementary Table 1, implying that the extracted Mooney-Rivlin parameters correctly describe the real material behavior.


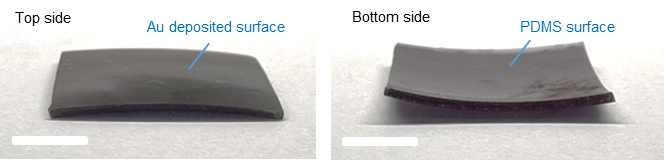


**Suppl. Figure 13. Differential tangential expansion of Au-PDMS nanophase.** Digital photos of the Au deposited PDMS (*φ* = 3.5) membrane after the gyrification for 6 hr. The top side shows a convex shape where the Au deposited surface is facing up, and the bottom side shows a concave shape where the PDMS surface is facing up. The PDMS membrane was 1.25 mm in thick, 3 cm in width and 2.5 cm in height, and the Au deposition rate was 2.5 Å s^-1^. The images show the convex appearances of a sample that was aged over 6 h on a PDMS pad (length 3 cm, width 2.5 cm, and thickness 1.25 mm). The convex shape is due to the expansion of the deposited surface. Scale bar is 1 cm.


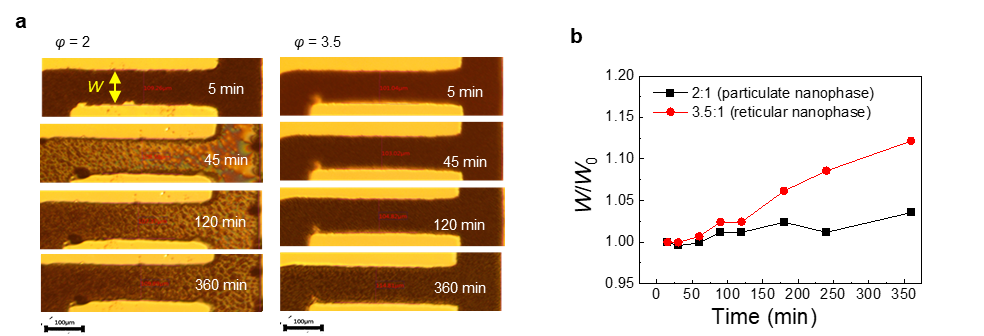


**Suppl. Figure 14. Differential tangential expansion of Au-PDMS nanophase with various *φ*.** **a**, Optical microscopy images (yellow color *W* is an electrode pattern width) and **b,** relative change of *W* over time (e.g., gyrification) for particulate (*φ* = 2) and reticular nanophase (*φ* = 3.5). Using the metal shadow mask, 100 nm of gold at a rate of 2.5 Å s^‑1^ was deposited on PDMS (*φ* = 3.5). The line width of the metal mask was 100 µm, and the line width of the obtained electrode pattern was 140 µm.

**Suppl. Figure 15. Long-term stability.** The conductivity of gyrified Au-PDMS nanophase sample remained almost constant for years. Error bars indicate the standard deviation of three independent measurements. All the standard deviations are within 4453 Scm^-1^, and the difference between all samples is smaller than this standard deviation value.


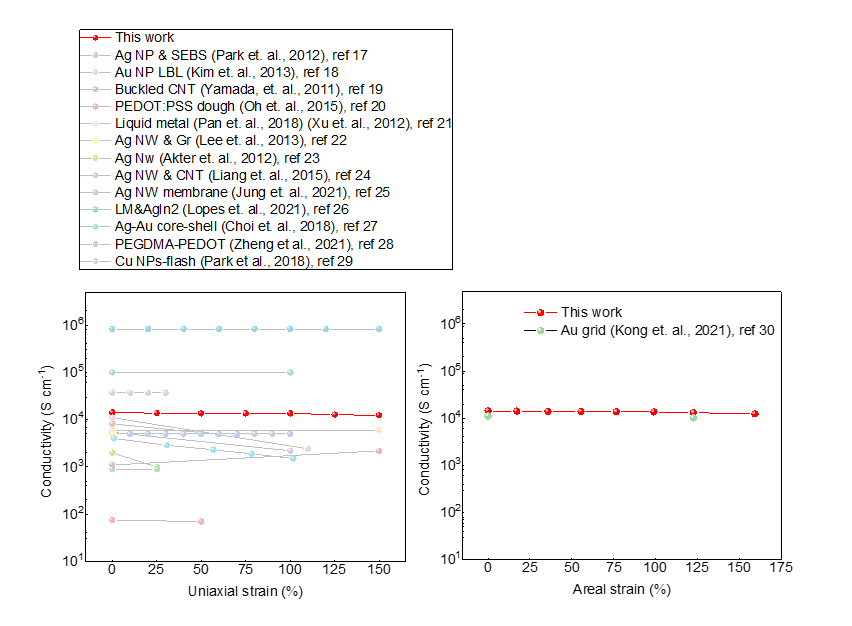


**Suppl. Figure 16. Outstanding performance of Au-PDMS nanophase in terms of strain invariant conductivity.** Compared strain-dependent conductivity (left for uniaxial- and right for areal-) of the gyrified Au-PDMS nanophases across the stretchable conductors previously reported (refs.^17–30^).


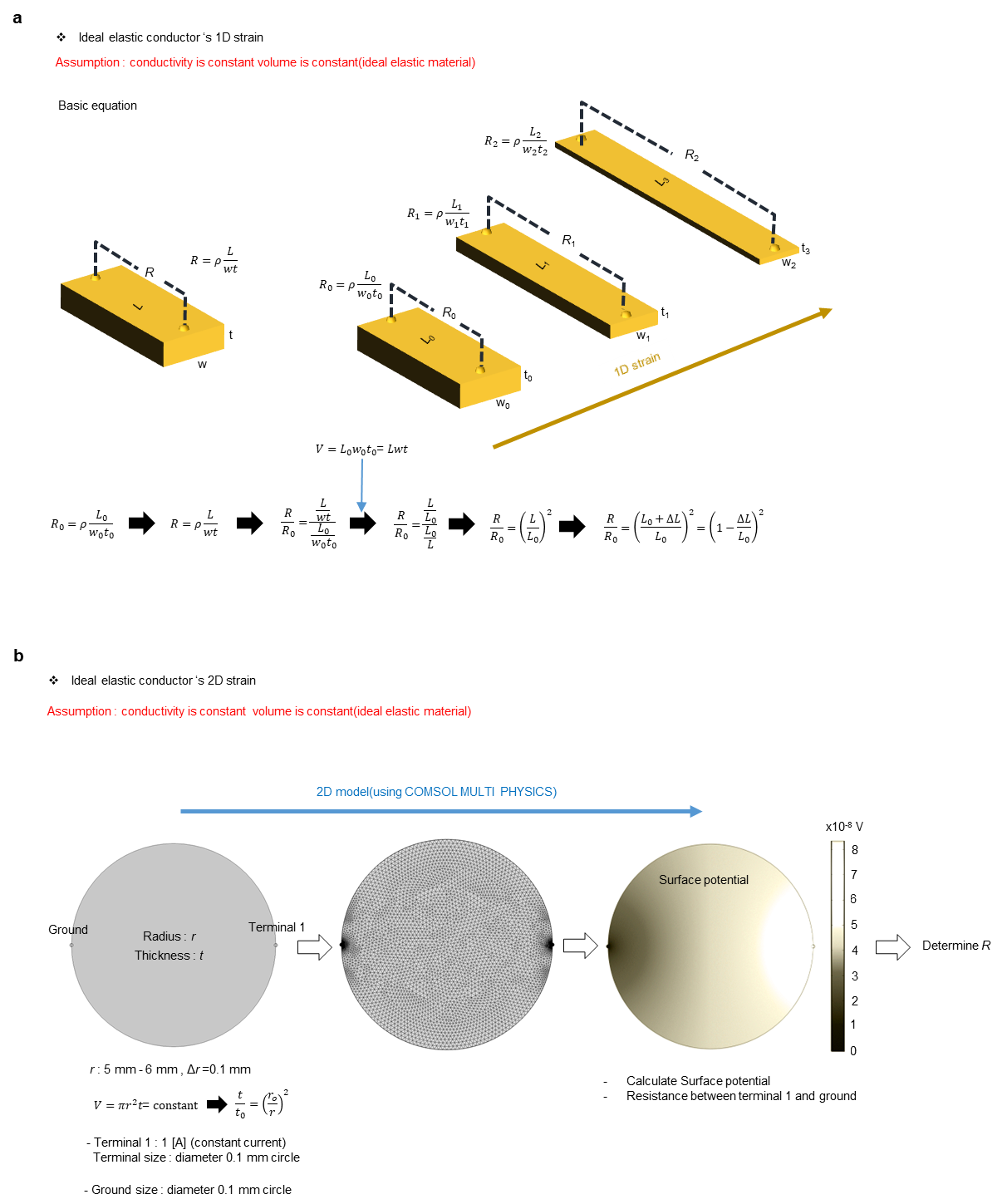


**Suppl. Figure 17. Investigation of strain dependence resistance of Au-PDMS nanophase.** Calculation of ideal elastic conductor upon **a**, uniaxial and **b**, areal strain.


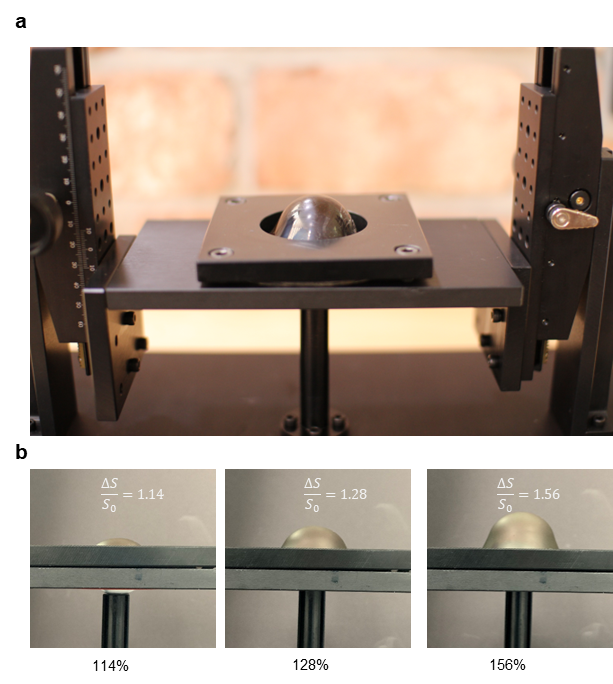


**Suppl. Figure 18. Characterization of the Au-PDMS nanophase upon area strain by custom made bulge test equipment.** **a**, Digital photos of the equipment. **b**, Calculation of area strain of Au-PDMS nanophase.


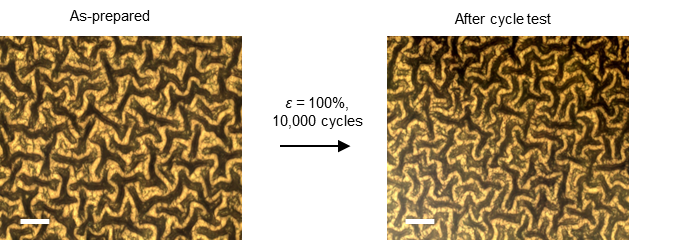


**Suppl. Figure 19. Mechanical stability of the gyrified Au-PDMS reticular nanophase sample.** Up to 10,000 times of stretching cycle for 100% strain, the surface morphology of the stretchable Au-PDMS nanophase showed no change, implying that the wrinkle has remarkable durable and stable mechanical stability (no delamination, no crack formation). Scale bars denote 10 μm.


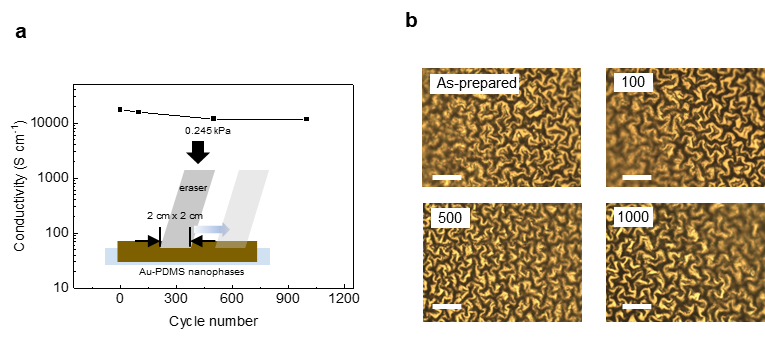


**Suppl. Figure 20. Mechanical durability. a**, Abrasion test by cyclic eraser, showing highly durable conductivity of the sample. **b**, Optical microscopy images after each cyclic eraser test. Scale bar denotes 20 μm. Numbers represent the number of cycles of mechanical rubbing.


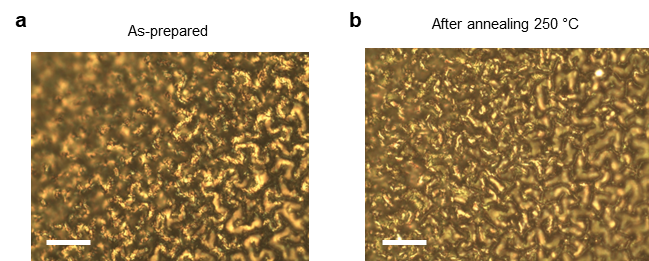


**Suppl. Figure 21. Thermal stability of the gyrified Au-PDMS nanophases.** Optical microscopy images of the sample before **a**, and after **b**, annealing at 250 °C for 2 h.


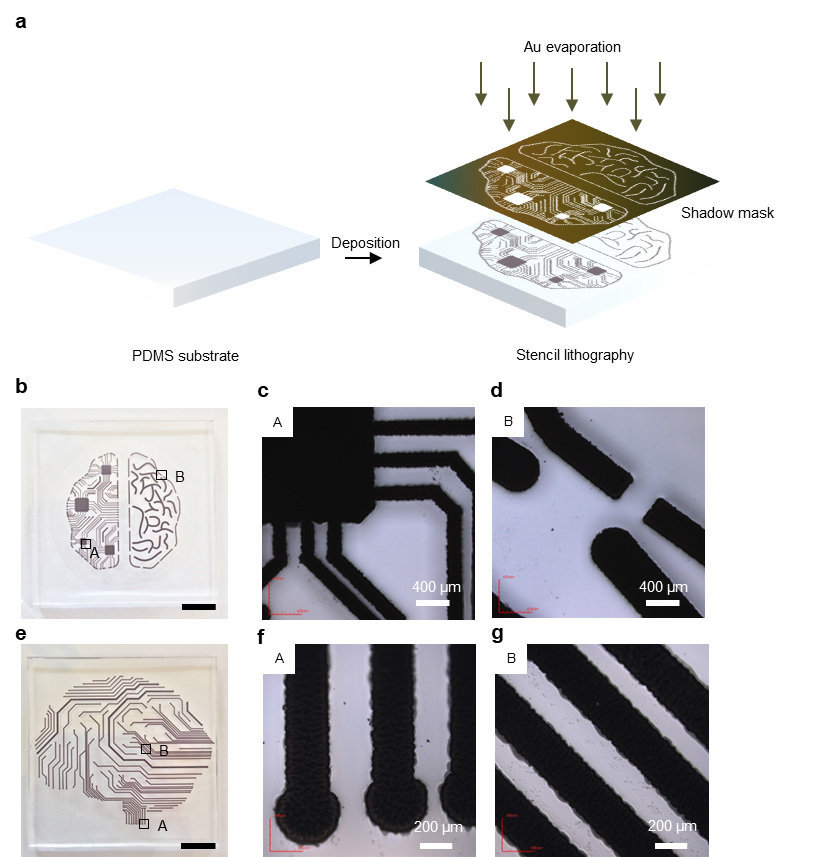


**Suppl. Figure 22. Stencil mask lithography.** Stencil mask lithography of the gyrified Au-PDMS reticular nanophases sample, displaying ability of high-resolution pattering of the soft conductor. **a**, Scheme for metal-elastomer electrode patterning using stencil lithography. Scale bar denotes 0.5 cm. **b**,**e**, Photographs of the patterned electrode with various shadow mask. **c**,**d**,**f**,**g**, Optical microscopy images of each parts in the electrode assigned to the corresponding alphabet ‘A’, ‘B’ in **b**, **e**.


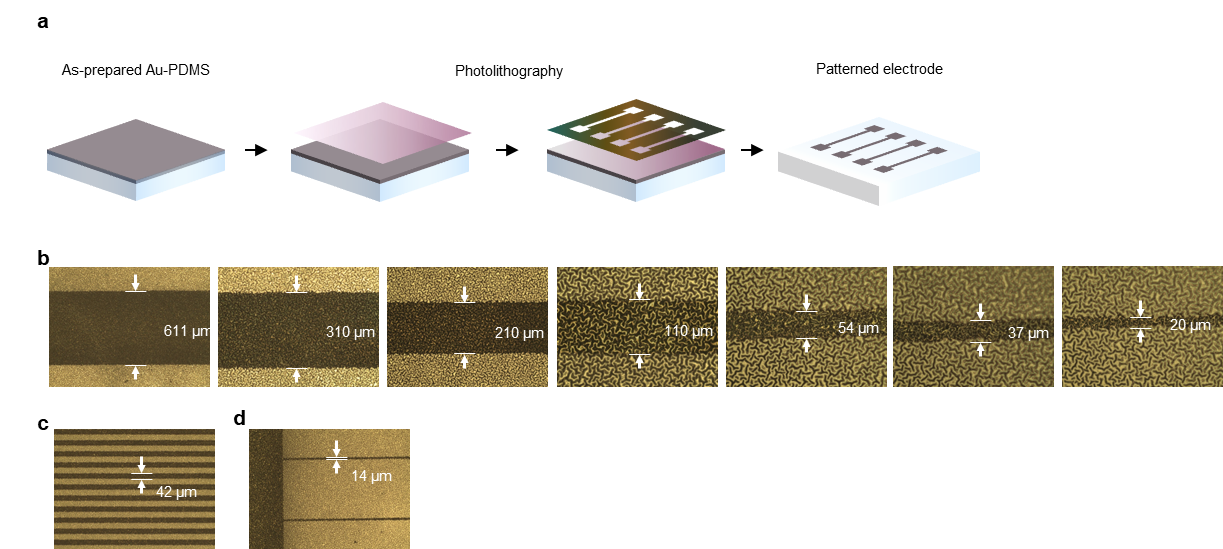


**Suppl. Figure 23. Photolithography of gyrified Au-PDMS reticular nanophase.** The chemical and mechanical durability of the 3D structure of Au-PDMS nanophases sample enables us to create patterns for stretchable conductors using conventional techniques. **a**, Scheme for metal-elastomer electrode patterning using conventional photo lithography. Optical microscopy images of the patterned electrode up to **b**, 20 μm, **c**, 42 μm, **d**, 14 μm.


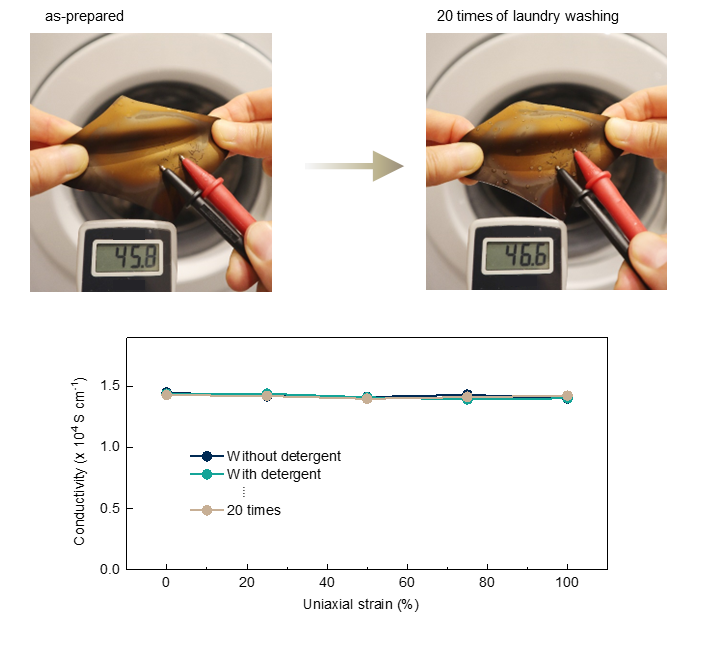


**Suppl. Figure 24. Laundry machine washable test** (see Suppl. Movie 5). Up to 20 times of washing (for each 15 min) by laundry machine, the stretchable conductivity (up to 100 % of uniaxial strain) showed no change, implying that the wrinkle sample could be used for durable and washable e-skin device even without any encapsulation.


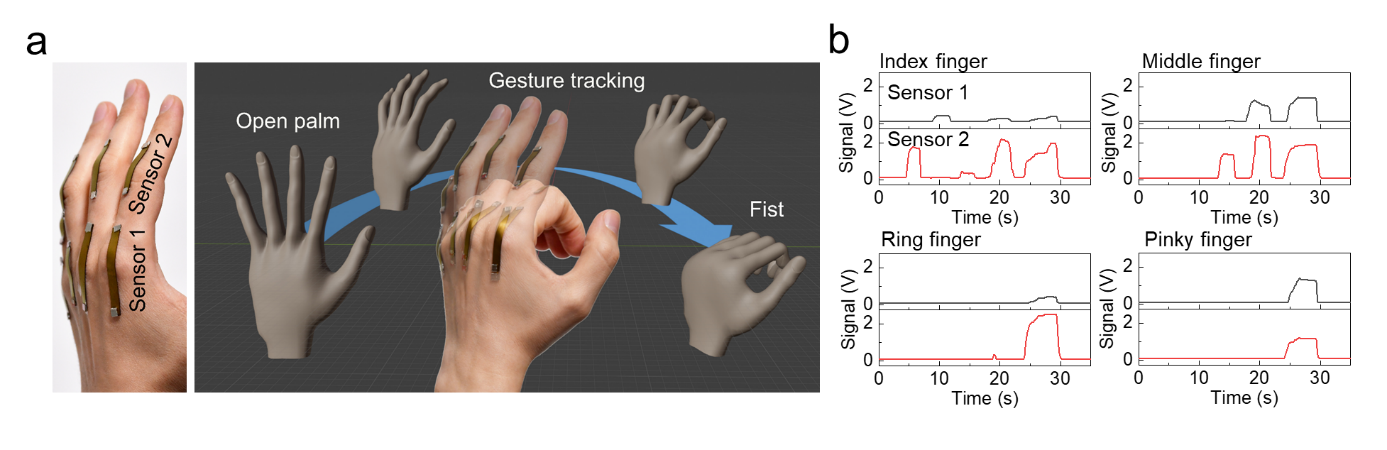


**Suppl. Figure 25. VR demonstration of the 3D complex of Au–PDMS nanophases.** **a,** Use of the 3D complex of Au–PDMS nanophases for gesture reconstruction in VR applications. Left: Photograph showing stretchable conductors mounted on finger joints. Each finger is decorated with two sensing elements. Right: Schematic representation of gesture tracking of a clenched fist in VR. **b,** Changes in electrical resistance of 3D complex of Au–PDMS nanophases in response to a sensor tracking the closing of fingers. Electrical response of stretchable conductors measured during the motion tracking of individual fingers is shown in Suppl. Movie 6 and Suppl. Note 8.


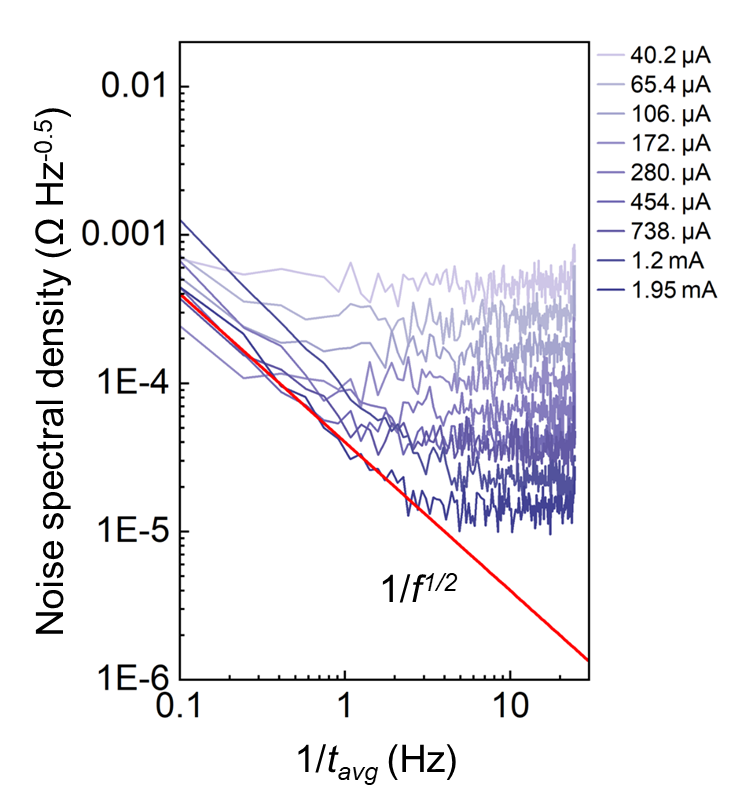


**Suppl. Figure 26. Electrical noise characterization of the Au-PDMS nanophase.** A set of noise spectral density plots measured for Au-PDMS nanophase wrinkled sample at different driving currents. The noise spectra show clear 1/√𝑓 trend with base noise density of 20 Ω / √Hz and cutoff frequency of 2.8 Hz.

**Suppl. Table 1.** Hyperelastic constants *C*_ij_ of plain PDMS membrane of *φ* = 3.5, obtained by the strain-stress curve. Component ratio means the weight ratio of prepolymer PDMS to crosslinker.

| Component ratio | *C*_10_ (Pa) | *C*_01_ (Pa) | *C*_20_ (Pa) | *C*_02_ (Pa) | *C* _11_ (Pa) |
| --- | --- | --- | --- | --- | --- |
| 3.5:1 | $-6.30\times{10}^{6}$ | $-7.12\times{10}^{6}$ | $8.48\times{10}^{6}$ | $2.27\times{10}^{7}$ | $-2.56\times{10}^{7}$ |

**Suppl. Table 2.** Comparison table of various stretchable conductors with various material types (Carbons, Metals, Liquid Metals, and Mechanically-guided structural designs) previously reported in literature, mainly focusing on a greater stretchability and conductivity (Supplementary Fig. 1).

| Type | | Materials | Stretchability | | | | Conducting property (S,Ω) | | | | Reference | |
| --- | --- | --- | --- | --- | --- | --- | --- | --- | --- | --- | --- | --- |
|  |  |  | 1D | | 2D | |  |  |  |  |  |  |
| Carbon | | SWNT | 70% | | - | | 10 S cm^-1^ | | Ref. ^31^ | | | |
|  |  | SWNT | 405% | | - | | - | | Ref. ^32^ | | | |
|  |  | MWNT | 638% | | - | | - | | Ref. ^32^ | | | |
|  |  | CNT | 60% | | - | | ≈ 10 Ω □^-1^ | | Ref. ^33^ | | | |
|  |  | PDA-CNT | 700% | | - | | 8.2 S m^-1^ | | Ref. ^34^ | | | |
|  |  | c-MWCNT | 2761% | | - | | ≈1 S m^-1^ | | Ref. ^35^ | | | |
|  |  | N-MWCNT | 100% | | - | | - | | Ref. ^36^ | | | |
|  |  | Carbon fibers | - | | - | | 20,000 S m^-1^ | | Ref. ^37^ | | | |
|  |  | Carbon fibers | 7.9% | | - | | - | | Ref. ^38^ | | | |
|  |  | GO | 40% | | - | | 1,660 Ω cm^-1^ | | Ref. ^39^ | | | |
|  |  | SWCNT | 3.10% | | - | | 300 S cm^-1^ | | Ref. ^40^ | | | |
|  |  | CNT/Graphene | 100% | | - | | 0.01 S cm^-1^ | | Ref. ^41^ | | | |
|  |  | CNT | 70% | | - | | 1 MΩ @ 5 Hz | | Ref. ^42^ | | | |
|  |  | PDA-pGO | 1700% | | - | | 0.08 S cm^-1^ | | Ref. ^43^ | | | |
|  |  | CNT | - | | - | | 0.0672 S m^-1^ | | Ref. ^44^ | | | |
|  |  | CNT | 100% | | - | | 1.7 Ω □^-1^ | | Ref. ^45^ | | | |
|  |  | Aligned-CNT ribbon/PDMS | 100% | | - | | 18.8 kΩ | | Ref. ^46^ | | | |
|  |  | Double-layer graphene | 36.2% | | - | | - | | Ref. ^47^ | | | |
|  |  | Graphene–AgNWs hybrid | 80% | | - | | 1 Ω □^-1^ | | Ref. ^48^ | | | |
|  |  | MWCNT-PDMS | 45% | | - | | 0.1 S cm^-1^ | | Ref. ^49^ | | | |
|  |  | MWCNT-PDMS | 50% | | - | | 10^-3^ S cm^-1^ | | Ref. ^50^ | | | |
|  |  | MWCNT-PDMS | 40% | | - | | 6.5×10^−2^ S cm^-1^ | | Ref. ^51^ | | | |
|  |  | Graphene–AgNWs hybrid | 100% | | - | | 33 Ω □^-1^ | | Ref. ^52^ | | | |
|  |  | SWCNTs/fluorinated copolymer composite | >100% | | - | | ≈100 S cm^-1^ | | Ref. ^53^ | | | |
|  |  | CNT–PMIA core–shell | 150% | | - | | 109 S cm^-1^ | | Ref ^54^ | | | |
|  |  | Carbon-black/styrene butadiene composite | 200% | | - | | 40 S cm^-1^ | | Ref. ^55^ | | | |
|  |  | CNT/R-GO/porous PDMS | 50% | | - | | 27 S m^-1^ | | Ref. ^56^ | | | |
|  |  | R-GO microtubes/PDMS | 50% | | - | | - | | Ref. ^57^. | | | |
|  |  | R-GO/nanocellulose | 100% | | - | | - | | Ref. ^58^. | | | |
|  |  | GNP-w-CNT | 150% | | - | | 132 S m^-1^ | | Ref. ^59^ | | | |
| Type | Materials | | | Stretchability | | | | Conductivity | | | | Reference |
|  |  |  |  | 1D | | 2D | |  |  |  |  |  |
| Metal | AgNW | | | 40% | | - | | - | | Ref. ^60^ | | |
|  | AgNW | | | 400% | | - | | 100 Ω □^-1^ | | Ref. ^61^ | | |
|  | AuNP | | | 40% | | - | | - | | Ref. ^43^ | | |
|  | Au-TiO_2_NW | | | 150% | | - | | 16,000 S cm^-1^, 10 kΩ @ 1 kHz | | Ref. ^62^ | | |
|  | AuNW-AgF | | | 1900% | | - | | 70 S cm^-1^ | | Ref. ^63^ | | |
|  | AgNW | | | 90% | | - | | 11,210 S cm^-1^ | | Ref. ^64^ | | |
|  | AgNW | | | 25% | | - | | 1.9 Ω □^-1^ | | Ref. ^65^ | | |
|  | AgNWs | | | 20% | | - | | 35 Ω □^-1^ | | Ref. ^66^ | | |
|  | CuNWs | | | 17% | | - | | 220 Ω □^-1^ | | Ref. ^67^ | | |
|  | AgNWs/PDMS | | | 50% | | - | | 7.5 Ω □^-1^ | | Ref. ^68^ | | |
|  | AgNWs/PDMS | | | 20% | | - | | 9 Ω □^-1^ | | Ref. ^69^ | | |
|  | AgNWs/PUU/PDMS | | | 50% | | - | | 5 – 425 Ω □^-1^ | | Ref. ^70^ | | |
|  | AgNWs/PDMS | | | 30% | | - | | 26.1 Ω □^-1^ | | Ref. ^71^ | | |
|  | AgNWs/PDMS | | | 50% | | - | | - | | Ref. ^72^ | | |
|  | Silver salt/polystyrene-block-polyisoprene-block-polystyrene | | | 200% | | - | | 0.8 Ω □^-1^ | | Ref. ^73^ | | |
|  | Ni/PDMS composite | | | 100% | | - | | 20 Ω | | Ref. ^74^ | | |
|  | Ni electroplated onto porous PDMS surface | | | 80% | | - | | - | | Ref. ^75^ | | |
|  | AgNP/SWCNT/PU composite | | | 90% | | - | | 620 S cm^-1^ | | Ref. ^76^ | | |
|  | AgNWs/PEDOT:PSS/PU | | | 130% | | - | | 36 Ω □^-1^ | | Ref. ^77^ | | |
|  | AgNW/TPU | | | 600% | | - | | 5,000 S cm^-1^ | | Ref. ^78^ | | |
|  | AgNW | | | 50% | | - | | 8130 S cm^-1^ | | Ref. ^79^ | | |
|  | Ag flake | | | 400% | | - | | 6168 S cm^-1^ | | Ref. ^80^ | | |
|  | Au atom | | | 130% | | - | | ≈50 Ω | | Ref. ^81^ | | |
|  | AgF | | | 250% | | - | | 374 S cm^-1^ | | Ref. ^82^ | | |
|  | AuNP | | | 486% | | - | | 6800 S cm^-1^ | | Ref. ^18^ | | |
|  | AgNP | | | 1000% | | - | | 0.05 S cm^-1^ | | Ref. ^83^ | | |
|  | AgNW | | | 100% | | - | | ≈0.8 Ω | | Ref. ^84^ | | |
|  | Ag-AuNW | | | 840% | | - | | 72,600 S cm^-1^ | | Ref. ^85^ | | |
|  | CuNW | | | - | | - | | ≈54 Ω □^-1^ | | Ref. ^86^ | | |
|  | AgF | | | 1780% | | - | | 501 Ω | | Ref. ^87^ | | |
|  | Ag-AuNW | | | 720% | | - | | 29,500 S cm^-1^, 112 Ω (@ 1kHz) | | Ref. ^88^ | | |
|  | Ag flakes-PU | | | 600% | | - | | 3.6×10^3^ S cm^-1^ | | Ref. ^89^ | | |
|  | AgNW-PUA | | | 70% | | - | | 4.5×10^4^ S cm^-1^ | | Ref. ^90^ | | |
|  | AgNW-PDMS | | | 100% | | - | | 9.97×10^3^ S cm^-1^ | | Ref. ^91^ | | |
|  | AgNW-PDMS | | | 15% | | - | | 8.13×10^3^ S cm^-1^ | | Ref. ^92^ | | |
|  | Ag flakes-PU | | | 74% | | - | | 4.31×10^4^ S cm^-1^ | | Ref. ^93^ | | |

| Type | Materials | Stretchability | | Conductivity | | Reference |
| --- | --- | --- | --- | --- | --- | --- |
|  |  | 1D | 2D |  |  |  |
| Liquid Metal | EGaIn/PDMS | 500% | - | 2,316 S cm^-1^ | Ref. ^94^ | |
|  | EGaIn/PDMS | 50% | - | 1370 S cm^-1^ | Ref. ^95^ | |
|  | EGaIn | 400% | - | ≈1 Ω | Ref. ^96^ | |
|  | Biphasic Ga-In | 1,000% | - | 2.06 × 10^6^ S m^-1^ | Ref. ^97^ | |
|  | EGaIn/polyphenols | 70% | - | 1.6 × 10^6^ S m^-1^ | Ref. ^98^ | |
|  | EGaIn/SEBS | 250% | - | 46 Ω □^-1^ | Ref. ^99^ | |
|  | EGaIn/LAPONITE^®^ | 150% | - | ≈ 10^5^ S m^-1^ | Ref. ^100^ | |
|  | EGaIn | 100% | - | 64 Ω | Ref. ^101^ | |
|  | EGaIn | 680% | - | 0.05 Ω | Ref. ^102^ | |
|  | EGaIn/SEBS | 800% | - | 3.4 × 10^4^ S cm^-1^ | Ref. ^103^ | |
|  | GaInSn | 50% | - | - | Ref. ^104^ | |

| Type | Materials | Stretchability | | Conductivity | | Reference |
| --- | --- | --- | --- | --- | --- | --- |
|  |  | Uniaxial strain | Areal strain |  |  |  |
| Mechanically-guided  structural designs | GaAs/Si | 100% | - | - | Ref. ^105^ | |
|  | SWNT | 30% | - | - | Ref. ^106^ | |
|  | Si | 15% | - | - | Ref. ^107^ | |
|  | Si | 140% | - | - | Ref. ^108^ | |
|  | Si | 4% | - | - | Ref. ^109^ | |
|  | Au | 14.2% | - | - | Ref. ^110^ | |
|  | Si | 100% | - | - | Ref. ^111^ | |

*Hyphen (-) means that the reference has no information about the factors

**Suppl. Table 3.** Comparison of durability performance of the Au-PDMS nanophases with previously reported stretchable conductors (Supplementary Note 8)

| Materials | Electrical property | Stretchability | | Durability | | | | Ref. |
| --- | --- | --- | --- | --- | --- | --- | --- | --- |
|  |  | Uniaxial (1D) strain | Areal (2D) strain | pH | Thermal | Solvent | Washing |  |
| Au nanophase-3D complex | 14200 S cm^-1^ | 12290 S/cm @150%  (F/I:98%) | 12140S/cm @156% | 2 - 13 | up to 250 °C | Water, Ethanol, Acetone,  Chlorobenzene, Toluene | Laundry machine (20 × 15 min) with detergent | This work |
| Cu Zr metallic glasses | 3.8 Ω □^-1^ | 4.94 Ω/sq  **@**70% | - | **-** | 180 °C | **-** | **-** | Ref. ^112^ |
| PAA, PDMAPS, IL | 0.01 S cm^-1^ | -  **@**1000% | - | 7 | 100 °C | **-** | **-** | Ref. ^113^ |
| Ag, MWCNT, PDMS | 6450 S cm^-1^ | 3745 S/cm  **@**120% | - | **-** | **-** | **-** | Laundry machine  (10 s) | Ref. ^114^ |
| MWCNTs, m-PDMS | 86.33 Ω □^-1^ | -  **@**30% | - | 0 - 13 | **-** | Ethanol | **-** | Ref. ^115^ |
| Cu/Pu fiber | 10^5^ S cm^-1^ | ≈10^5^ S/cm  **@**200% | - | **-** | 20 °C | Water | Ultrasonic  (100 min) | Ref. ^116^ |
| Cu/Ecoflex | 0.4 Ω | -  **@**100% | - | **-** | **-** | Water | Laundry machine  (30 min with detergent) | Ref. ^117^ |
| rGO/PEDOT:PSS | 153 Ω □^-1^ | -  **@**60% | - | **-** | 80 °C | **-** | Hand washing  (15 cycles, 30 min at 45 °C, drying 10 min at 80 °C) | Ref. ^118^ |
| Ag NWs/TPU | 5114 S □^-1^ | 50.89 S/cm  **@** 600% | - | **-** | 120 °C | **-** | Simulated laundry washing  (10 min, drying at 60 °C for 30 min) | Ref. ^78^ |
| AgMFs | 0.2 Ω □^-1^ | 6.6 Ω/sq  **@**160% | - | **-** | 209.4 °C | **-** | **-** | Ref. ^119^ |
| Ag NPs | 24.8 Ω | ≈50 Ω  **@**50% | - | 2 - 12 | 118.7 °C | Water | **-** | Ref. ^120^ |
| PEDOT/Mxene | 3.6 Ω □^-1^ | - | - | **-** | 193.1 °C | **-** | **-** | Ref. ^121^ |
| rGO | 24.7 Ω □^-1^ | 148.2 Ω/sq  **@**20% | - | **-** | 138 °C | **-** | **-** | Ref. ^122^ |
| PEDOT:PSS/EG | 74 S cm^-1^ | -  **@**10% | - | **-** | **-** | **--** | Laundry machine (15 cycles, 40 min at 30 °C with detergent) | Ref. ^123^ |
| Au coated fabric | 1.07 Ω □^-1^ | 0.214 Ω/sq  **@**160% | - | 0.9 g/L NaCl, 0.2 g/L KCl | **-** | **-** | Stirring for 1h, drying at 60 °C overnight | Ref. ^124^ |
| Au-PI | **-** | **@**53% | - | 5 - 9 | **-** | Sweat equivalent solution | **-** | Ref. ^125^ |
| Au-PI (TiSi_2_) | 2.5 Ω □^-1^ | - | - | 7.4 (PBS)  for 10 days | 96 °C | **-** | **-** | Ref. ^126^ |
| PANI-EB/Laser carbonized PI | 0.065 Ω cm^-1^ | 0.13 Ω/cm  **@**144% | - | 4 - 10 | **-** | PBS | **-** | Ref. ^127^ |
| Au NS | 1.4 Ω □^-1^ | 1.554 Ω/sq  **@**50% | - | 4 - 8 | **-** | **-** | **-** | Ref. ^128^ |
| Nanoporous Au | **-** | **@**30% | - | 5 – 7 | **-** | **-** | **-** | Ref. ^129^ |
|  |  |  |  |  |  |  |  |  |

*Hyphen (-) means that the reference has no information about the factors

*F/I means a ratio of final electrical property and initial electrical property

**Suppl. References**

1. Cheng, X. *et al.* Controlled fabrication of nanoscale wrinkle structure by fluorocarbon plasma for highly transparent triboelectric nanogenerator. *Microsyst Nanoeng* **3**, 16074 (2017).

2. Kim, M., Moon, B. U. & Hidrovo, C. H. Enhancement of the thermo-mechanical properties of PDMS molds for the hot embossing of PMMA microfluidic devices. *J. Micromech. Microeng.* **23**, (2013).

3. Matei, A., Ţucureanu, V. & Popescu, M. Synthesis and characterization of various surfactants for stabilized CuO powder. *Powder Metal Adv Materi* **8**, 52–60 (2018).

4. Choi, W. J. *et al.* Terahertz circular dichroism spectroscopy of biomaterials enabled by kirigami polarization modulators. *Nat Mater* **18**, 820–826 (2019).

5. Khanafer, K., Duprey, A., Schlicht, M. & Berguer, R. Effects of strain rate, mixing ratio, and stress–strain definition on the mechanical behavior of the polydimethylsiloxane (PDMS) material as related to its biological applications. *Biomed Microdevices* **11**, 503–508 (2009).

6. Seghir, R. & Arscott, S. Extended PDMS stiffness range for flexible systems. *Sens Actuators A Phys* **230**, 33–39 (2015).

7. Bîrsan, M., Ghiba, I.-D., Martin, R. J. & Neff, P. Refined dimensional reduction for isotropic elastic Cosserat shells with initial curvature. *Math. Mech. Solids* **24**, 4000–4019 (2019).

8. Sander, O. Geodesic finite elements of higher order. IMA J. Numer. Anal. 38, 1, 238-266 (2015) doi:10.1093/imanum/drv016.

9. Sander, O., Neff, P. & Bîrsan, M. Numerical treatment of a geometrically nonlinear planar Cosserat shell model. *Comput Mech* **57**, 817–841 (2016).

10. Sander, O. *DUNE — The Distributed and Unified Numerics Environment*. vol. 140 Springer International Publishing, 2020.

11. Guo, Q. *et al.* 3D multi-stable structures with surface wrinkling patterns. *Surf Coat Technol* **416**, (2021).

12. Kim, E. S. *et al.* Facile fabrication of micro/nano-structured wrinkles by controlling elastic properties of polydimethylsiloxane substrates. *Polymer* **212**, (2021).

13. Wei, Y. *et al.* Liquid metal fillers enabled remote actuating and localizing reversible wrinkles on polymeric bilayer. *Appl Mater Today* **28**, 101537 (2022).

14. Bowden, N., Brittain, S., Evans, A. G., Hutchinson, J. W. & Whitesides, G. M. Spontaneous formation of ordered structures in thin films of metals supported on an elastomeric polymer. *Nature* **393**, 146–149 (1998).

15. Lacour, S. P., Wagner, S., Huang, Z. & Suo, Z. Stretchable gold conductors on elastomeric substrates. *Appl Phys Lett* **82**, 2404–2406 (2003).

16. Kim, J. *et al.* Highly stretchable wrinkled gold thin film wires. *Appl Phys Lett* **108**, (2016).

17. Park, M. *et al.* Highly stretchable electric circuits from a composite material of silver nanoparticles and elastomeric fibres. *Nat Nanotechnol* **7**, 803–809 (2012).

18. Kim, Y. *et al.* Stretchable nanoparticle conductors with self-organized conductive pathways. *Nature* **500**, 59–63 (2013).

19. Yamada, T. *et al.* A stretchable carbon nanotube strain sensor for human-motion detection. *Nat Nanotechnol* **6**, 296–301 (2011).

20. Oh, J. Y., Kim, S., Baik, H. & Jeong, U. Conducting Polymer Dough for Deformable Electronics. *Adv Mater* **28**, 4455–4461 (2016).

21. Pan, C. *et al.* Visually Imperceptible Liquid‐Metal Circuits for Transparent, Stretchable Electronics with Direct Laser Writing. *Adv Mater* **30**, (2018).

22. Lee, D. *et al.* Highly stable and flexible silver nanowire–graphene hybrid transparent conducting electrodes for emerging optoelectronic devices. *Nanoscale* **5**, 7750 (2013).

23. Akter, T. & Kim, W. S. Reversibly Stretchable Transparent Conductive Coatings of Spray-Deposited Silver Nanowires. *ACS Appl Mater Interfaces* **4**, 1855–1859 (2012).

24. Liang, J. *et al.* Intrinsically stretchable and transparent thin-film transistors based on printable silver nanowires, carbon nanotubes and an elastomeric dielectric. *Nat Commun* **6**, 7647 (2015).

25. Jung, D. *et al.* Highly conductive and elastic nanomembrane for skin electronics. *Science* **373**, 1022–1026 (2021).

26. Lopes, P. A., Santos, B. C., de Almeida, A. T. & Tavakoli, M. Reversible polymer-gel transition for ultra-stretchable chip-integrated circuits through self-soldering and self-coating and self-healing. *Nat Commun* **12**, 4666 (2021).

27. Choi, S. *et al.* Highly conductive, stretchable and biocompatible Ag–Au core–sheath nanowire composite for wearable and implantable bioelectronics. *Nat Nanotechnol* **13**, 1048–1056 (2018).

28. Zheng, Y.-Q. *et al.* Monolithic optical microlithography of high-density elastic circuits. *Science* **373**, 88–94 (2021).

29. Park, J. H. *et al.* Flash‐Induced Stretchable Cu Conductor via Multiscale‐Interfacial Couplings. *Adv Sci* **5**, (2018).

30. Kong, M. *et al.* Transparent Omni‐Directional Stretchable Circuit Lines Made by a Junction‐Free Grid of Expandable Au Lines. *Adv Mater* **33**, (2021).

31. Sekitani, T. *et al.* A Rubberlike Stretchable Active Matrix Using Elastic Conductors. *Science* **321**, 1468–1472 (2008).

32. Xia, H. & Song, M. Preparation and characterization of polyurethane–carbon nanotube composites. *Soft Matter* **1**, 386 (2005).

33. Kim, J. H. *et al.* Simple and cost-effective method of highly conductive and elastic carbon nanotube/polydimethylsiloxane composite for wearable electronics. *Sci Rep* **8**, 1375 (2018).

34. Han, L. *et al.* Mussel‐Inspired Adhesive and Conductive Hydrogel with Long‐Lasting Moisture and Extreme Temperature Tolerance. *Adv Funct Mater* **28**, (2018).

35. Xia, S., Song, S., Jia, F. & Gao, G. A flexible, adhesive and self-healable hydrogel-based wearable strain sensor for human motion and physiological signal monitoring. *J Mater Chem B* **7**, 4638–4648 (2019).

36. Fu, X. *et al.* Hydrogel Cryo‐Microtomy Continuously Making Soft Electronic Devices. *Adv Funct Mater* **31**, (2021).

37. Khosla, A. *et al.* Carbon fiber doped thermosetting elastomer for flexible sensors: physical properties and microfabrication. *Sci Rep* **8**, 12313 (2018).

38. Wang, B. *et al.* High-Performance, Biobased, Degradable Polyurethane Thermoset and Its Application in Readily Recyclable Carbon Fiber Composites. *ACS Sustain Chem Eng* **8**, 11162–11170 (2020).

39. Shi, G. *et al.* A versatile PDMS submicrobead/graphene oxide nanocomposite ink for the direct ink writing of wearable micron-scale tactile sensors. *Appl Mater Today* **16**, 482–492 (2019).

40. Liang, L., Gao, C., Chen, G. & Guo, C.-Y. Large-area, stretchable, super flexible and mechanically stable thermoelectric films of polymer/carbon nanotube composites. *J Mater Chem C Mater* **4**, 526–532 (2016).

41. Kim, T., Park, J., Sohn, J., Cho, D. & Jeon, S. Bioinspired, Highly Stretchable, and Conductive Dry Adhesives Based on 1D–2D Hybrid Carbon Nanocomposites for All-in-One ECG Electrodes. *ACS Nano* **10**, 4770–4778 (2016).

42. Hoon Lee, J. *et al.* CNT/PDMS-based canal-typed ear electrodes for inconspicuous EEG recording. *J Neural Eng* **11**, 046014 (2014).

43. You, I. *et al.* Stretchable E‐Skin Apexcardiogram Sensor. *Adv Mater* **28**, 6359–6364 (2016).

44. Nam, J. *et al.* Supramolecular Peptide Hydrogel-Based Soft Neural Interface Augments Brain Signals through a Three-Dimensional Electrical Network. *ACS Nano* **14**, 664–675 (2020).

45. Hong, S. *et al.* Stretchable Electrode Based on Laterally Combed Carbon Nanotubes for Wearable Energy Harvesting and Storage Devices. *Adv Funct Mater* **27**, (2017).

46. Zhang, Y. *et al.* Polymer‐Embedded Carbon Nanotube Ribbons for Stretchable Conductors. *Adv Mater* **22**, 3027–3031 (2010).

47. Won, S. *et al.* Double-layer CVD graphene as stretchable transparent electrodes. *Nanoscale* **6**, 6057–6064 (2014).

48. An, B. W. *et al.* Stretchable and Transparent Electrodes using Hybrid Structures of Graphene–Metal Nanotrough Networks with High Performances and Ultimate Uniformity. *Nano Lett* **14**, 6322–6328 (2014).

49. Ha-Chul Jung *et al.* CNT/PDMS Composite Flexible Dry Electrodesfor Long-Term ECG Monitoring. *IEEE Trans Biomed Eng* **59**, 1472–1479 (2012).

50. Lee, J.-B. & Khang, D.-Y. Electrical and mechanical characterization of stretchable multi-walled carbon nanotubes/polydimethylsiloxane elastomeric composite conductors. *Compos Sci Technol* **72**, 1257–1263 (2012).

51. Liu, C.-X. & Choi, J.-W. Patterning conductive PDMS nanocomposite in an elastomer using microcontact printing. *J. Micromech. Microeng.* **19**, 085019 (2009).

52. Lee, M.-S. *et al.* High-Performance, Transparent, and Stretchable Electrodes Using Graphene–Metal Nanowire Hybrid Structures. *Nano Lett* **13**, 2814–2821 (2013).

53. Sekitani, T. *et al.* Stretchable active-matrix organic light-emitting diode display using printable elastic conductors. *Nat Mater* **8**, 494–499 (2009).

54. Vural, M., Behrens, A. M., Ayyub, O. B., Ayoub, J. J. & Kofinas, P. Sprayable Elastic Conductors Based on Block Copolymer Silver Nanoparticle Composites. *ACS Nano* **9**, 336–344 (2015).

55. Bhagavatheswaran, E. S. *et al.* Construction of an Interconnected Nanostructured Carbon Black Network: Development of Highly Stretchable and Robust Elastomeric Conductors. *J. Phys. Chem. C* **119**, 21723–21731 (2015).

56. Chen, M. *et al.* Highly Stretchable Conductors Integrated with a Conductive Carbon Nanotube/Graphene Network and 3D Porous Poly(dimethylsiloxane). *Adv Funct Mater* **24**, 7548–7556 (2014).

57. Tang, Y. *et al.* Highly Stretchable and Ultrasensitive Strain Sensor Based on Reduced Graphene Oxide Microtubes–Elastomer Composite. *ACS Appl Mater Interfaces* **7**, 27432–27439 (2015).

58. Yan, C. *et al.* Highly Stretchable Piezoresistive Graphene–Nanocellulose Nanopaper for Strain Sensors. *Adv Mater* **26**, 2022–2027 (2014).

59. Zhang, F. *et al.* 3D Interconnected Conductive Graphite Nanoplatelet Welded Carbon Nanotube Networks for Stretchable Conductors. *Adv Funct Mater* **31**, (2021).

60. Ho, M. D. *et al.* Percolating Network of Ultrathin Gold Nanowires and Silver Nanowires toward “Invisible” Wearable Sensors for Detecting Emotional Expression and Apexcardiogram. *Adv Funct Mater* **27**, (2017).

61. Huang, G.-W., Xiao, H.-M. & Fu, S.-Y. Wearable Electronics of Silver-Nanowire/Poly(dimethylsiloxane) Nanocomposite for Smart Clothing. *Sci Rep* **5**, 13971 (2015).

62. Tybrandt, K. *et al.* High‐Density Stretchable Electrode Grids for Chronic Neural Recording. *Adv Mater* **30**, (2018).

63. Seo, H. *et al.* Durable and Fatigue‐Resistant Soft Peripheral Neuroprosthetics for In Vivo Bidirectional Signaling. *Adv Mater* **33**, (2021).

64. Park, J. *et al.* Electromechanical cardioplasty using a wrapped elasto-conductive epicardial mesh. *Sci Transl Med* **8**, (2016).

65. Li, W., Yang, S. & Shamim, A. Screen printing of silver nanowires: balancing conductivity with transparency while maintaining flexibility and stretchability. *npj Flex Electron* **3**, 13 (2019).

66. Akter, T. & Kim, W. S. Reversibly Stretchable Transparent Conductive Coatings of Spray-Deposited Silver Nanowires. *ACS Appl Mater Interfaces* **4**, 1855–1859 (2012).

67. Cheng, Y., Wang, S., Wang, R., Sun, J. & Gao, L. Copper nanowire based transparent conductive films with high stability and superior stretchability. *J. Mater. Chem. C* **2**, 5309–5316 (2014).

68. Hu, W. *et al.* Intrinsically stretchable transparent electrodes based on silver-nanowire–crosslinked-polyacrylate composites. *Nanotechnology* **23**, 344002 (2012).

69. Cheng, T. *et al.* High-performance stretchable transparent electrodes based on silver nanowires synthesized via an eco-friendly halogen-free method. *J. Mater. Chem. C* **2**, 10369–10376 (2014).

70. Kim, D.-H., Yu, K.-C., Kim, Y. & Kim, J.-W. Highly Stretchable and Mechanically Stable Transparent Electrode Based on Composite of Silver Nanowires and Polyurethane–Urea. *ACS Appl Mater Interfaces* **7**, 15214–15222 (2015).

71. Park, S.-M. *et al.* Metal nanowire percolation micro-grids embedded in elastomers for stretchable and transparent conductors. *J Mater Chem C Mater* **3**, 8241–8247 (2015).

72. Yao, S. & Zhu, Y. Wearable multifunctional sensors using printed stretchable conductors made of silver nanowires. *Nanoscale* **6**, 2345 (2014).

73. Vural, M., Behrens, A. M., Ayyub, O. B., Ayoub, J. J. & Kofinas, P. Sprayable Elastic Conductors Based on Block Copolymer Silver Nanoparticle Composites. *ACS Nano* **9**, 336–344 (2015).

74. Kim, S. *et al.* Negatively Strain‐Dependent Electrical Resistance of Magnetically Arranged Nickel Composites: Application to Highly Stretchable Electrodes and Stretchable Lighting Devices. *Adv Mater* **26**, 3094–3099 (2014).

75. Jeong, G. S. *et al.* Solderable and electroplatable flexible electronic circuit on a porous stretchable elastomer. *Nat Commun* **3**, 977 (2012).

76. Ki, H. *et al.* Chemically Driven, Water-Soluble Composites of Carbon Nanotubes and Silver Nanoparticles as Stretchable Conductors. *ACS Macro Lett* **4**, 769–773 (2015).

77. Hwang, B.-U. *et al.* Transparent Stretchable Self-Powered Patchable Sensor Platform with Ultrasensitive Recognition of Human Activities. *ACS Nano* **9**, 8801–8810 (2015).

78. Zhao, H. *et al.* Ultrastretchable and Washable Conductive Microtextiles by Coassembly of Silver Nanowires and Elastomeric Microfibers for Epidermal Human–Machine Interfaces. *ACS Mater Lett* **3**, 912–920 (2021).

79. Xu, F. & Zhu, Y. Highly Conductive and Stretchable Silver Nanowire Conductors. *Adv Mater* **24**, 5117–5122 (2012).

80. Matsuhisa, N. *et al.* Printable elastic conductors by in situ formation of silver nanoparticles from silver flakes. *Nat Mater* **16**, 834–840 (2017).

81. Liu, Z. *et al.* Highly Stable and Stretchable Conductive Films through Thermal‐Radiation‐Assisted Metal Encapsulation. *Adv Mater* **31**, (2019).

82. Ohm, Y. *et al.* An electrically conductive silver–polyacrylamide–alginate hydrogel composite for soft electronics. *Nat Electron* **4**, 185–192 (2021).

83. Shin, M., Song, K. H., Burrell, J. C., Cullen, D. K. & Burdick, J. A. Injectable and Conductive Granular Hydrogels for 3D Printing and Electroactive Tissue Support. *Adv Sci* **6**, (2019).

84. Choi, S. *et al.* Stretchable Heater Using Ligand-Exchanged Silver Nanowire Nanocomposite for Wearable Articular Thermotherapy. *ACS Nano* **9**, 6626–6633 (2015).

85. Choi, S. *et al.* Highly conductive, stretchable and biocompatible Ag–Au core–sheath nanowire composite for wearable and implantable bioelectronics. *Nat Nanotechnol* **13**, 1048–1056 (2018).

86. Yin, Z. *et al.* Curved copper nanowires-based robust flexible transparent electrodes via all-solution approach. *Nano Res* **10**, 3077–3091 (2017).

87. Kim, S. H. *et al.* Ultrastretchable Conductor Fabricated on Skin‐Like Hydrogel–Elastomer Hybrid Substrates for Skin Electronics. *Adv Mater* **30**, (2018).

88. Sunwoo, S. *et al.* Stretchable Low‐Impedance Nanocomposite Comprised of Ag–Au Core–Shell Nanowires and Pt Black for Epicardial Recording and Stimulation. *Adv Mater Technol* **5**, (2020).

89. Araki, T., Nogi, M., Suganuma, K., Kogure, M. & Kirihara, O. Printable and Stretchable Conductive Wirings Comprising Silver Flakes and Elastomers. *IEEE Electron Device Lett* **32**, 1424–1426 (2011).

90. Liang, J., Tong, K. & Pei, Q. A Water‐Based Silver‐Nanowire Screen‐Print Ink for the Fabrication of Stretchable Conductors and Wearable Thin‐Film Transistors. *Adv Mater* **28**, 5986–5996 (2016).

91. Martinez, V. *et al.* Stretchable Silver Nanowire–Elastomer Composite Microelectrodes with Tailored Electrical Properties. *ACS Appl Mater Interfaces* **7**, 13467–13475 (2015).

92. Song, L., Myers, A. C., Adams, J. J. & Zhu, Y. Stretchable and Reversibly Deformable Radio Frequency Antennas Based on Silver Nanowires. *ACS Appl Mater Interfaces* **6**, 4248–4253 (2014).

93. Suikkola, J. *et al.* Screen-Printing Fabrication and Characterization of Stretchable Electronics. *Sci Rep* **6**, 25784 (2016).

94. Tang, L. *et al.* Printable Metal-Polymer Conductors for Highly Stretchable Bio-Devices. *iScience* **4**, 302–311 (2018).

95. Markvicka, E. J., Bartlett, M. D., Huang, X. & Majidi, C. An autonomously electrically self-healing liquid metal–elastomer composite for robust soft-matter robotics and electronics. *Nat Mater* **17**, 618–624 (2018).

96. Chen, X. *et al.* Self-healing and stretchable conductor based on embedded liquid metal patterns within imprintable dynamic covalent elastomer. *J Mater Chem C Mater* **10**, 1039–1047 (2022).

97. Liu, S., Shah, D. S. & Kramer-Bottiglio, R. Highly stretchable multilayer electronic circuits using biphasic gallium-indium. *Nat Mater* **20**, 851–858 (2021).

98. Rahim, Md. A. *et al.* Polyphenol‐Induced Adhesive Liquid Metal Inks for Substrate‐Independent Direct Pen Writing. *Adv Funct Mater* **31**, (2021).

99. Martin‐Monier, L., Gupta, T. Das, Yan, W., Lacour, S. & Sorin, F. Nanoscale Controlled Oxidation of Liquid Metals for Stretchable Electronics and Photonics. *Adv Funct Mater* **31**, (2021).

100. Wu, P., Zhou, L., Lv, S., Fu, J. & He, Y. Self-sintering liquid metal ink with LAPONITE® for flexible electronics. *J Mater Chem C Mater* **9**, 3070–3080 (2021).

101. Boley, J. W., White, E. L., Chiu, G. T. ‐C. & Kramer, R. K. Direct Writing of Gallium‐Indium Alloy for Stretchable Electronics. *Adv Funct Mater* **24**, 3501–3507 (2014).

102. Wang, H. *et al.* A Highly Stretchable Liquid Metal Polymer as Reversible Transitional Insulator and Conductor. *Adv Mater* **31**, (2019).

103. Zhu, S. *et al.* Ultrastretchable Fibers with Metallic Conductivity Using a Liquid Metal Alloy Core. *Adv Funct Mater* **23**, 2308–2314 (2013).

104. Matsuzaki, R. & Tabayashi, K. Highly Stretchable, Global, and Distributed Local Strain Sensing Line Using GaInSn Electrodes for Wearable Electronics. *Adv Funct Mater* **25**, 3806–3813 (2015).

105. Sun, Y., Choi, W. M., Jiang, H., Huang, Y. Y. & Rogers, J. A. Controlled buckling of semiconductor nanoribbons for stretchable electronics. *Nat Nanotechnol* **1**, 201–207 (2006).

106. Yu, C., Masarapu, C., Rong, J., Wei, B. & Jiang, H. Stretchable Supercapacitors Based on Buckled Single‐Walled Carbon‐Nanotube Macrofilms. *Adv Mater* **21**, 4793–4797 (2009).

107. Jiang, H. *et al.* Finite deformation mechanics in buckled thin films on compliant supports. Proc Natl Acad Sci **104**, 15607–15612 (2007).

108. Kim, D.-H. *et al.* Materials and noncoplanar mesh designs for integrated circuits with linear elastic responses to extreme mechanical deformations. Proc Natl Acad Sci **105**, 18675–18680 (2008).

109. Choi, W. M. *et al.* Biaxially Stretchable “Wavy” Silicon Nanomembranes. *Nano Lett* **7**, 1655–1663 (2007).

110. Gray, D. S., Tien, J. & Chen, C. S. High‐Conductivity Elastomeric Electronics. *Adv Mater* **16**, 393–397 (2004).

111. Kim, D. *et al.* Ultrathin Silicon Circuits With Strain‐Isolation Layers and Mesh Layouts for High‐Performance Electronics on Fabric, Vinyl, Leather, and Paper. *Adv Mater* **21**, 3703–3707 (2009).

112. An, B. W. *et al.* Stretchable, Transparent Electrodes as Wearable Heaters Using Nanotrough Networks of Metallic Glasses with Superior Mechanical Properties and Thermal Stability. *Nano Lett* **16**, 471–478 (2016).

113. Lei, Z. & Wu, P. A highly transparent and ultra-stretchable conductor with stable conductivity during large deformation. *Nat Commun* **10**, 3429 (2019).

114. Ko, Y. *et al.* Stretchable Conductive Adhesives with Superior Electrical Stability as Printable Interconnects in Washable Textile Electronics. *ACS Appl Mater Interfaces* **11**, 37043–37050 (2019).

115. Zhong, X., Hu, H. & Fu, H. Self-Cleaning, Chemically Stable, Reshapeable, Highly Conductive Nanocomposites for Electrical Circuits and Flexible Electronic Devices. *ACS Appl Mater Interfaces* **10**, 25697–25705 (2018).

116. Yang, Z. *et al.* Conductive and Elastic 3D Helical Fibers for Use in Washable and Wearable Electronics. *Adv Mater* **32**, (2020).

117. Zhao, Y. *et al.* Scaling Metal‐Elastomer Composites toward Stretchable Multi‐Helical Conductive Paths for Robust Responsive Wearable Health Devices. *Adv Healthc Mater* **10**, (2021).

118. Ahmed, A. *et al.* A PEDOT:PSS and graphene-clad smart textile-based wearable electronic Joule heater with high thermal stability. *J Mater Chem C Mater* **8**, 16204–16215 (2020).

119. Kim, Y. Il *et al.* Highly transparent, conducting, body-attachable metallized fibers as a flexible and stretchable film. *J Alloys Compd* **790**, 1127–1136 (2019).

120. Guo, Z. *et al.* Multi-functional and water-resistant conductive silver nanoparticle-decorated cotton textiles with excellent joule heating performances and human motion monitoring. *Cellulose* **28**, 7483–7495 (2021).

121. Zheng, X. *et al.* Vapor phase polymerized conducting polymer/MXene textiles for wearable electronics. *Nanoscale* **13**, 1832–1841 (2021).

122. Wang, D., Li, D., Zhao, M., Xu, Y. & Wei, Q. Multifunctional wearable smart device based on conductive reduced graphene oxide/polyester fabric. *Appl Surf Sci* **454**, 218–226 (2018).

123. Lund, A. *et al.* Roll‐to‐Roll Dyed Conducting Silk Yarns: A Versatile Material for E‐Textile Devices. *Adv Mater Technol* **3**, (2018).

124. Wu, Y., Mechael, S. S., Chen, Y. & Carmichael, T. B. Solution Deposition of Conformal Gold Coatings on Knitted Fabric for E‐Textiles and Electroluminescent Clothing. *Adv Mater Technol* **3**, (2018).

125. Dang, W. *et al.* Stretchable wireless system for sweat pH monitoring. *Biosens Bioelectron* **107**, 192–202 (2018).

126. Li, J. *et al.* Ultrathin, Transferred Layers of Metal Silicide as Faradaic Electrical Interfaces and Biofluid Barriers for Flexible Bioelectronic Implants. *ACS Nano* **13**, 660–670 (2019).

127. Rahimi, R. *et al.* Highly Stretchable Potentiometric pH Sensor Fabricated via Laser Carbonization and Machining of Carbon−Polyaniline Composite. *ACS Appl Mater Interfaces* **9**, 9015–9023 (2017).

128. Oh, S. Y. *et al.* Skin-Attachable, Stretchable Electrochemical Sweat Sensor for Glucose and pH Detection. *ACS Appl Mater Interfaces* **10**, 13729–13740 (2018).

129. Bae, C. W. *et al.* Fully Stretchable Capillary Microfluidics-Integrated Nanoporous Gold Electrochemical Sensor for Wearable Continuous Glucose Monitoring. *ACS Appl Mater Interfaces* **11**, 14567–14575 (2019).
