# Supplementary material for: Kinetically controlled metal-elastomer nanophases for environmentally resilient stretchable electronics
Source: Nat Commun. 2024 Apr 9;15:3071. doi: 10.1038/s41467-024-47223-6 (PMC11004024; doi:10.1038/s41467-024-47223-6)
Supplement: Supplementary file 3 — Description of Additional Supplementary Files [file 41467_2024_47223_MOESM3_ESM.pdf]

**Description of Additional Supplementary Files**

File Name: Supplementary Movie 1
Description: In-situ gyrification of 3D structure of Au-PDMS nanophases by Au 100 nm thick deposition on the PDMS ($\varphi = 3.5$) membrane. Scale bar denotes 30 μm.

File Name: Supplementary Movie 2
Description: 3D TEM tomography movie of Au-PDMS nanophase, showing the interconnected and interpenetrated Au-PDMS nanophase and microscale surface 3D structure.

File Name: Supplementary Movie 3
Description: Eraser test of 3D structure of Au-PDMS nanophases sample

File Name: Supplementary Movie 4
Description: In-situ thermal annealing test, showing superior thermal durability of the 3D structure of Au-PDMS nanophases sample up to 250 °C.

File Name: Supplementary Movie 5
Description: Washing test by laundry machine with detergent.

File Name: Supplementary Movie 6
Description: Virtual reality demonstration using Au-PDMS nanophase.
